# Supplementary material for: Divergent Pairwise Epistasis in the Context of Unstable Membrane Protein Variants
Source: bioRxiv. 2023 Aug 26:2023.08.25.554866. Preprint. [Version 1] doi: 10.1101/2023.08.25.554866 (PMC10473758; doi:10.1101/2023.08.25.554866)
Supplement: Supplementary file 1 [file media-1.pdf]

Supplementary Information

Divergent Pairwise Epistasis in the Context of Unstable Membrane Protein Variants

Laura M. Chamness,[1] Charles P. Kuntz,[2] Andrew G. McKee,[1] Wesley D. Penn,[1] Christopher M. Hemmerich,[3] Douglas B. Rusch,[3] Hope Woods,[4,5] Dyotima,[1] Jens Meiler,[4,6] and Jonathan P. Schlebach[1,2*]

[1]*Department of Chemistry, Indiana University, Bloomington, Indiana, USA*
[2]*Department of Chemistry, Purdue University, West Lafayette, Indiana, USA*
[3]*Center for Genomics and Bioinformatics, Indiana University, Bloomington, Indiana, USA*
[4]*Department of Chemistry, Vanderbilt University, Nashville, Tennessee, USA*
[5]*Chemical and Physical Biology Program, Vanderbilt University, Nashville, Tennessee, USA*
[6]*Institute for Drug Development, Leipzig University, Leipzig, SAC, Germany*

**Corresponding author* E-mail address: jschleba@purdue.edu

This Document Includes:
-Figure S1
-Figure S2
-Table S1
-Table S2
-Table S3

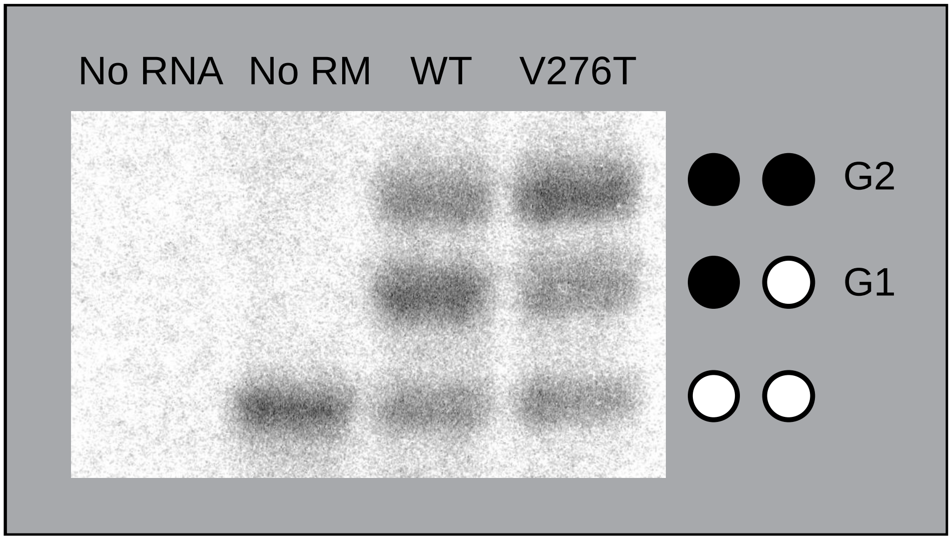

Figure S1. Membrane integration of mGnRHR TM domain 6 and V276T. Chimeric Lep proteins containing either the wild type mGnRHR TM domain 6 or the V276T mutant, with the guest TM domain flanked by two glycosylation sites, were translated *in vitro* and analyzed by SDS-PAGE. Misintegration of the guest TM domain results in doubly glycosylated protein, while successful membrane integration results in singly glycosylated protein. Negative control translation reactions without RNA (first lane) and without rough microsomes (second lane) are included. The positions of the untargeted protein that is not associated with the membrane (no glycosylation), singly glycosylated form of the protein in which TMD6 is integrated into the membrane (G1), and doubly glycosylated form of the protein in which TMD6 is not integrated into the membrane (G2) are indicated for reference.

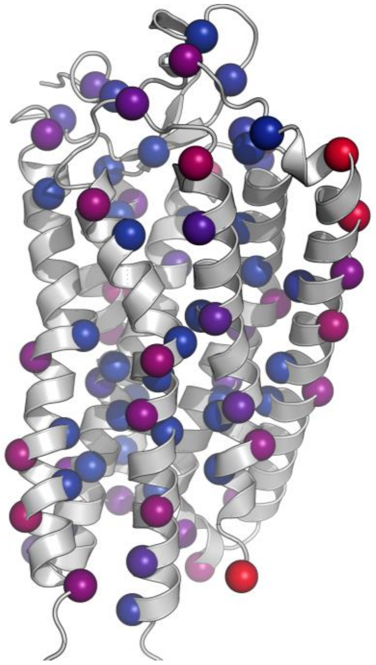

Figure S2. Structural context of mGnRHR library mutants. Residues selected for mutation are displayed as spheres on the homology model of *M. musculus* GnRHR. Spheres are colored according to solvent accessible surface area of the residue, with red representing exposed residues and blue representing buried residues.

Table S1. Predicted and measured apparent transfer free energies of mGnRHR TM6.

Insert TMD	K_{app} ‡ ($Int._{G1}$ / $Int._{G2}$)	ΔG_{app} ‡ (kcal/ mol)	$\Delta G_{app, pred}$ † (kcal/ mol)
WT TMD6	1.2 ± 0.2	-0.1 ± 0.1	0.26
V276T TMD6	0.7 ± 0.1	0.2 ± 0.1	0.805

‡Values represent the average of three experimental replicates, and errors represent the standard deviation.
†Values predicted from the TM6 sequence using the ΔG Predictor.

Table S2. Solvent accessible surface area of mGnRHR residues.

Position	Residue	Percent Relative Accessibility (REL)
13	His	91.1
14	Cys	63.9
15	Ser	21.9
16	Ala	32.1
17	Ile	67.3
18	Asn	76.5
19	Asn	1.3
20	Ser	15.6
21	Ile	3.9
22	Pro	27.2
23	Leu	3.2
24	Ile	50.7
25	Gln	33.3
26	Gly	9.0
27	Lys	69.8
28	Leu	7.6
29	Pro	44.6
30	Thr	45.0
31	Leu	13.5
32	Thr	84.7
33	Val	69.0
34	Ser	13.8
35	Gly	4.5
36	Lys	80.6
37	Ile	51.7
38	Arg	7.0
39	Val	29.3
40	Thr	49.0
41	Val	31.7
42	Thr	12.3
43	Phe	59.6
44	Phe	62.2
45	Leu	3.5
46	Phe	27.5
47	Leu	64.6
48	Leu	31.0
49	Ser	0.0
50	Thr	31.0

51	Ala	51.6
52	Phe	29.7
53	Asn	0.0
54	Ala	30.6
55	Ser	40.6
56	Phe	7.0
57	Leu	11.6
58	Leu	57.0
59	Lys	54.6
60	Leu	0.7
61	Gln	44.7
62	Lys	72.6
63	Trp	26.1
64	Thr	49.3
65	Gln	81.5
66	Lys	53.8
67	Arg	91.8
68	Lys	59.2
69	Lys	34.7
70	Gly	24.7
71	Lys	71.5
72	Lys	42.9
73	Leu	3.7
74	Ser	10.8
75	Arg	18.0
76	Met	4.0
77	Lys	7.5
78	Val	9.2
79	Leu	0.1
80	Leu	2.2
81	Lys	21.5
82	His	14.5
83	Leu	1.5
84	Thr	0.0
85	Leu	33.8
86	Ala	0.2
87	Asn	6.0
88	Leu	17.4
89	Leu	37.1
90	Glu	4.4
91	Thr	0.1

92	Leu	52.1
93	Ile	44.3
94	Val	3.0
95	Met	3.0
96	Pro	49.0
97	Leu	16.9
98	Asp	3.0
99	Gly	16.4
100	Met	27.2
101	Trp	14.8
102	Asn	5.0
103	Ile	21.9
104	Thr	43.7
105	Val	5.6
106	Gln	12.7
107	Trp	0.5
108	Tyr	57.0
109	Ala	17.3
110	Gly	49.6
111	Glu	36.5
112	Phe	69.9
113	Leu	34.1
114	Cys	0.2
115	Lys	21.2
116	Val	11.2
117	Leu	9.3
118	Ser	14.0
119	Tyr	24.0
120	Leu	30.6
121	Lys	3.3
122	Leu	5.6
123	Phe	23.2
124	Ser	6.1
125	Met	6.5
126	Tyr	0.0
127	Ala	0.0
128	Pro	7.3
129	Ala	1.3
130	Phe	21.6
131	Met	0.3
132	Met	5.8

133	Val	11.5
134	Val	6.7
135	Ile	2.4
136	Ser	0.0
137	Leu	43.5
138	Asp	2.1
139	Arg	9.4
140	Ser	22.0
141	Leu	21.4
142	Ala	15.6
143	Ile	14.6
144	Thr	31.0
145	Gln	59.5
146	Pro	50.1
147	Leu	95.7
148	Ala	35.3
149	Val	67.0
150	Gln	65.6
151	Ser	4.0
152	Asn	41.5
153	Ser	40.8
154	Lys	85.6
155	Leu	40.3
156	Glu	2.4
157	Glm	40.2
158	Ser	41.0
159	Met	33.0
160	Ile	0.8
161	Ser	45.5
162	Leu	46.4
163	Ala	1.5
164	Trp	17.8
165	Ile	52.0
166	Leu	37.9
167	Ser	0.0
168	Ile	60.8
169	Val	58.1
170	Phe	24.9
171	Ala	4.4
172	Gly	45.4
173	Pro	23.8

174	Gln	3.7
175	Leu	40.7
176	Tyr	29.5
177	Ile	0.0
178	Phe	13.1
179	Arg	36.4
180	Met	21.9
181	Ile	7.9
182	Tyr	64.6
183	Leu	40.2
184	Ala	0.3
185	Asp	20.1
186	Gly	97.0
187	Ser	26.6
188	Gly	2.2
189	Pro	80.5
190	Thr	70.8
191	Val	63.0
192	Phe	11.8
193	Ser	2.2
194	Gln	5.3
195	Cys	9.0
196	Val	6.3
197	Thr	0.5
198	His	4.9
199	Cys	0.0
200	Ser	21.3
201	Phe	54.6
202	Pro	38.7
203	Gln	40.4
204	Trp	43.1
205	Trp	43.3
206	His	19.3
207	Gln	0.0
208	Ala	3.9
209	Phe	31.8
210	Tyr	26.2
211	Asn	0.7
212	Phe	57.2
213	Phe	46.1
214	Thr	5.6

215	Phe	19.7
216	Gly	19.0
217	Cys	24.8
218	Leu	4.1
219	Phe	6.3
220	Ile	34.9
221	Ile	50.2
222	Pro	7.7
223	Leu	20.8
224	Leu	52.4
225	Ile	36.8
226	Met	2.7
227	Leu	55.4
228	Ile	58.2
229	Cys	14.9
230	Asn	7.5
231	Ala	35.5
232	Lys	63.7
233	Ile	0.1
234	Ile	34.0
235	Phe	70.0
236	Ala	25.7
237	Leu	20.4
238	Thr	51.5
239	Arg	73.4
240	Val	42.9
241	Leu	74.1
242	His	96.1
243	Gln	64.9
244	Asp	23.5
245	Pro	44.3
246	Arg	67.7
247	Lys	51.7
248	Leu	41.1
249	Gln	46.3
250	Leu	51.1
251	Asn	26.2
252	Gln	76.8
253	Ser	71.5
254	Lys	61.6
255	Asn	93.2

256	Asn	43.8
257	Ile	55.2
258	Pro	12.6
259	Arg	72.2
260	Ala	39.4
261	Arg	29.7
262	Leu	31.1
263	Arg	8.3
264	Thr	13.3
265	Leu	4.2
266	Lys	55.6
267	Met	2.4
268	Thr	5.2
269	Val	39.8
270	Ala	39.5
271	Phe	3.3
272	Ala	13.6
273	Thr	51.0
274	Ser	9.2
275	Phe	6.0
276	Val	28.4
277	Val	57.2
278	Cys	1.4
279	Trp	2.4
280	Thr	17.6
281	Pro	20.2
282	Tyr	14.6
283	Tyr	6.5
284	Val	29.8
285	Leu	3.1
286	Gly	7.2
287	Ile	26.7
288	Trp	56.9
289	Tyr	18.0
290	Trp	23.1
291	Phe	35.9
292	Asp	51.7
293	Pro	14.0
294	Glu	81.8
295	Met	41.9
296	Leu	11.8

297	Asn	53.7
298	Arg	48.4
299	Val	65.5
300	Ser	16.8
301	Glu	3.0
302	Pro	17.3
303	Val	34.4
304	Asn	4.1
305	His	2.9
306	Phe	48.8
307	Phe	24.6
308	Phe	6.8
309	Leu	11.4
310	Phe	36.9
311	Ala	0.1
312	Phe	1.9
313	Leu	28.8
314	Asn	1.3
315	Pro	1.2
316	Cys	2.2
317	Phe	36.7
318	Asp	1.2
319	Pro	4.4
320	Leu	52.2
321	Ile	18.7
322	Tyr	0.8
323	Gly	31.6
324	Tyr	66.1
325	Phe	26.3
326	Ser	62.2
327	Leu	89.8

Table S3. mGnRHR library mutants with associated oligo sequences.

Mutation	Oligo Sequence
N4A	GGTACCATGGCTAACGCCGCATCTCTTGAGCAG
N4R	GGTACCATGGCTAACAGAGCATCTCTTGAGCAG
N4D	GGTACCATGGCTAACGACGCATCTCTTGAGCAG
N4C	GGTACCATGGCTAACTGCGCATCTCTTGAGCAG
N4Q	GGTACCATGGCTAACCAGGCATCTCTTGAGCAG
N4E	GGTACCATGGCTAACGAGGCATCTCTTGAGCAG
N4G	GGTACCATGGCTAACGGCGCATCTCTTGAGCAG
N4H	GGTACCATGGCTAACCACGCATCTCTTGAGCAG
N4I	GGTACCATGGCTAACATCGCATCTCTTGAGCAG
N4L	GGTACCATGGCTAACCTGGCATCTCTTGAGCAG
N4K	GGTACCATGGCTAACAAGGCATCTCTTGAGCAG
N4M	GGTACCATGGCTAACATGGCATCTCTTGAGCAG
N4F	GGTACCATGGCTAACTTCGCATCTCTTGAGCAG
N4P	GGTACCATGGCTAACCCCGCATCTCTTGAGCAG
N4S	GGTACCATGGCTAACAGCGCATCTCTTGAGCAG
N4T	GGTACCATGGCTAACACCGCATCTCTTGAGCAG
N4W	GGTACCATGGCTAACTGGGCATCTCTTGAGCAG
N4Y	GGTACCATGGCTAACTACGCATCTCTTGAGCAG
N4V	GGTACCATGGCTAACGTGGCATCTCTTGAGCAG
E8A	AACAATGCATCTCTTGCCCAGGACCCAAATCAC
E8R	AACAATGCATCTCTTAGACAGGACCCAAATCAC
E8N	AACAATGCATCTCTTAACCAGGACCCAAATCAC
E8D	AACAATGCATCTCTTGACCAGGACCCAAATCAC
E8C	AACAATGCATCTCTTTGCCAGGACCCAAATCAC
E8Q	AACAATGCATCTCTTCAGCAGGACCCAAATCAC
E8G	AACAATGCATCTCTTGGCCAGGACCCAAATCAC
E8H	AACAATGCATCTCTTCACCAGGACCCAAATCAC
E8I	AACAATGCATCTCTTATCCAGGACCCAAATCAC
E8L	AACAATGCATCTCTTCTGCAGGACCCAAATCAC
E8K	AACAATGCATCTCTTAAGCAGGACCCAAATCAC
E8M	AACAATGCATCTCTTATGCAGGACCCAAATCAC
E8F	AACAATGCATCTCTTTTCCAGGACCCAAATCAC
E8P	AACAATGCATCTCTTCCCCAGGACCCAAATCAC
E8S	AACAATGCATCTCTTAGCCAGGACCCAAATCAC
E8T	AACAATGCATCTCTTACCCAGGACCCAAATCAC
E8W	AACAATGCATCTCTTTGGCAGGACCCAAATCAC
E8Y	AACAATGCATCTCTTTACCAGGACCCAAATCAC
E8V	AACAATGCATCTCTTGTGCAGGACCCAAATCAC
N12A	CTTGAGCAGGACCCAGCCCACTGCTCGGCCATC

N12R	CTTGAGCAGGACCCAAGACACTGCTCGGCCATC
N12D	CTTGAGCAGGACCCAGACCACTGCTCGGCCATC
N12C	CTTGAGCAGGACCCATGCCACTGCTCGGCCATC
N12Q	CTTGAGCAGGACCCACAGCACTGCTCGGCCATC
N12E	CTTGAGCAGGACCCAGAGCACTGCTCGGCCATC
N12G	CTTGAGCAGGACCCAGGCCACTGCTCGGCCATC
N12H	CTTGAGCAGGACCCACACCACTGCTCGGCCATC
N12I	CTTGAGCAGGACCCAATCCACTGCTCGGCCATC
N12L	CTTGAGCAGGACCCACTGCACTGCTCGGCCATC
N12K	CTTGAGCAGGACCCAAAGCACTGCTCGGCCATC
N12M	CTTGAGCAGGACCCAATGCACTGCTCGGCCATC
N12F	CTTGAGCAGGACCCATTCCACTGCTCGGCCATC
N12P	CTTGAGCAGGACCCACCCCACTGCTCGGCCATC
N12S	CTTGAGCAGGACCCAAGCCACTGCTCGGCCATC
N12T	CTTGAGCAGGACCCAACCCACTGCTCGGCCATC
N12W	CTTGAGCAGGACCCATGGCACTGCTCGGCCATC
N12Y	CTTGAGCAGGACCCATACCACTGCTCGGCCATC
N12V	CTTGAGCAGGACCCAGTGCACTGCTCGGCCATC
A16R	CCAAATCACTGCTCGAGAATCAACAACAGCATC
A16N	CCAAATCACTGCTCGAACATCAACAACAGCATC
A16D	CCAAATCACTGCTCGGACATCAACAACAGCATC
A16C	CCAAATCACTGCTCGTGCATCAACAACAGCATC
A16Q	CCAAATCACTGCTCGCAGATCAACAACAGCATC
A16E	CCAAATCACTGCTCGGAGATCAACAACAGCATC
A16G	CCAAATCACTGCTCGGGCATCAACAACAGCATC
A16H	CCAAATCACTGCTCGCACATCAACAACAGCATC
A16I	CCAAATCACTGCTCGATCATCAACAACAGCATC
A16L	CCAAATCACTGCTCGCTGATCAACAACAGCATC
A16K	CCAAATCACTGCTCGAAGATCAACAACAGCATC
A16M	CCAAATCACTGCTCGATGATCAACAACAGCATC
A16F	CCAAATCACTGCTCGTTCATCAACAACAGCATC
A16P	CCAAATCACTGCTCGCCCATCAACAACAGCATC
A16S	CCAAATCACTGCTCGAGCATCAACAACAGCATC
A16T	CCAAATCACTGCTCGACCATCAACAACAGCATC
A16W	CCAAATCACTGCTCGTGGATCAACAACAGCATC
A16Y	CCAAATCACTGCTCGTACATCAACAACAGCATC
A16V	CCAAATCACTGCTCGGTGATCAACAACAGCATC
S20A	TCGGCCATCAACAACGCCATCCCCTTGATACAG
S20R	TCGGCCATCAACAACAGAATCCCCTTGATACAG
S20N	TCGGCCATCAACAACAACATCCCCTTGATACAG
S20D	TCGGCCATCAACAACGACATCCCCTTGATACAG

S20C	TCGGCCATCAACAACTGCATCCCCTTGATACAG
S20Q	TCGGCCATCAACAACCAGATCCCCTTGATACAG
S20E	TCGGCCATCAACAACGAGATCCCCTTGATACAG
S20G	TCGGCCATCAACAACGGCATCCCCTTGATACAG
S20H	TCGGCCATCAACAACCACATCCCCTTGATACAG
S20I	TCGGCCATCAACAACATCATCCCCTTGATACAG
S20L	TCGGCCATCAACAACCTGATCCCCTTGATACAG
S20K	TCGGCCATCAACAACAAGATCCCCTTGATACAG
S20M	TCGGCCATCAACAACATGATCCCCTTGATACAG
S20F	TCGGCCATCAACAACTTCATCCCCTTGATACAG
S20P	TCGGCCATCAACAACCCCATCCCCTTGATACAG
S20T	TCGGCCATCAACAACACCATCCCCTTGATACAG
S20W	TCGGCCATCAACAACTGGATCCCCTTGATACAG
S20Y	TCGGCCATCAACAACTACATCCCCTTGATACAG
S20V	TCGGCCATCAACAACGTGATCCCCTTGATACAG
I24A	AACAGCATCCCCTTGGCCCAGGGCAAGCTCCCG
I24R	AACAGCATCCCCTTGAGACAGGGCAAGCTCCCG
I24N	AACAGCATCCCCTTGAACCAGGGCAAGCTCCCG
I24D	AACAGCATCCCCTTGGACCAGGGCAAGCTCCCG
I24C	AACAGCATCCCCTTGTGCCAGGGCAAGCTCCCG
I24Q	AACAGCATCCCCTTGCAGCAGGGCAAGCTCCCG
I24E	AACAGCATCCCCTTGGAGCAGGGCAAGCTCCCG
I24G	AACAGCATCCCCTTGGGCCAGGGCAAGCTCCCG
I24H	AACAGCATCCCCTTGCACCAGGGCAAGCTCCCG
I24L	AACAGCATCCCCTTGCTGCAGGGCAAGCTCCCG
I24K	AACAGCATCCCCTTGAAGCAGGGCAAGCTCCCG
I24M	AACAGCATCCCCTTGATGCAGGGCAAGCTCCCG
I24F	AACAGCATCCCCTTGTTCCAGGGCAAGCTCCCG
I24P	AACAGCATCCCCTTGCCCCAGGGCAAGCTCCCG
I24S	AACAGCATCCCCTTGAGCCAGGGCAAGCTCCCG
I24T	AACAGCATCCCCTTGACCCAGGGCAAGCTCCCG
I24W	AACAGCATCCCCTTGTGGCAGGGCAAGCTCCCG
I24Y	AACAGCATCCCCTTGTACCAGGGCAAGCTCCCG
I24V	AACAGCATCCCCTTGGTGCAGGGCAAGCTCCCG
L28A	TTGATACAGGGCAAGGCCCCGACTCTAACCGTA
L28R	TTGATACAGGGCAAGAGACCGACTCTAACCGTA
L28N	TTGATACAGGGCAAGAACCCGACTCTAACCGTA
L28D	TTGATACAGGGCAAGGACCCGACTCTAACCGTA
L28C	TTGATACAGGGCAAGTGCCCGACTCTAACCGTA
L28Q	TTGATACAGGGCAAGCAGCCGACTCTAACCGTA
L28E	TTGATACAGGGCAAGGAGCCGACTCTAACCGTA

L28G	TTGATACAGGGCAAGGGCCCGACTCTAACCGTA
L28H	TTGATACAGGGCAAGCACCCGACTCTAACCGTA
L28I	TTGATACAGGGCAAGATCCCGACTCTAACCGTA
L28K	TTGATACAGGGCAAGAAGCCGACTCTAACCGTA
L28M	TTGATACAGGGCAAGATGCCGACTCTAACCGTA
L28F	TTGATACAGGGCAAGTTCCCGACTCTAACCGTA
L28P	TTGATACAGGGCAAGCCCCCGACTCTAACCGTA
L28S	TTGATACAGGGCAAGAGCCCGACTCTAACCGTA
L28T	TTGATACAGGGCAAGACCCCGACTCTAACCGTA
L28W	TTGATACAGGGCAAGTGGCCGACTCTAACCGTA
L28Y	TTGATACAGGGCAAGTACCCGACTCTAACCGTA
L28V	TTGATACAGGGCAAGGTGCCGACTCTAACCGTA
T32A	AAGCTCCCGACTCTAGCCGTATCTGGAAAGATC
T32R	AAGCTCCCGACTCTAAGAGTATCTGGAAAGATC
T32N	AAGCTCCCGACTCTAAACGTATCTGGAAAGATC
T32D	AAGCTCCCGACTCTAGACGTATCTGGAAAGATC
T32C	AAGCTCCCGACTCTATGCGTATCTGGAAAGATC
T32Q	AAGCTCCCGACTCTACAGGTATCTGGAAAGATC
T32E	AAGCTCCCGACTCTAGAGGTATCTGGAAAGATC
T32G	AAGCTCCCGACTCTAGGCGTATCTGGAAAGATC
T32H	AAGCTCCCGACTCTACACGTATCTGGAAAGATC
T32I	AAGCTCCCGACTCTAATCGTATCTGGAAAGATC
T32L	AAGCTCCCGACTCTACTGGTATCTGGAAAGATC
T32K	AAGCTCCCGACTCTAAAGGTATCTGGAAAGATC
T32M	AAGCTCCCGACTCTAATGGTATCTGGAAAGATC
T32F	AAGCTCCCGACTCTATTCGTATCTGGAAAGATC
T32P	AAGCTCCCGACTCTACCCGTATCTGGAAAGATC
T32S	AAGCTCCCGACTCTAAGCGTATCTGGAAAGATC
T32W	AAGCTCCCGACTCTATGGGTATCTGGAAAGATC
T32Y	AAGCTCCCGACTCTATACGTATCTGGAAAGATC
T32V	AAGCTCCCGACTCTAGTGGTATCTGGAAAGATC
K36A	CTAACCGTATCTGGAGCCATCCGAGTGACCGTG
K36R	CTAACCGTATCTGGAAGAATCCGAGTGACCGTG
K36N	CTAACCGTATCTGGAAACATCCGAGTGACCGTG
K36D	CTAACCGTATCTGGAGACATCCGAGTGACCGTG
K36C	CTAACCGTATCTGGATGCATCCGAGTGACCGTG
K36Q	CTAACCGTATCTGGACAGATCCGAGTGACCGTG
K36E	CTAACCGTATCTGGAGAGATCCGAGTGACCGTG
K36G	CTAACCGTATCTGGAGGCATCCGAGTGACCGTG
K36H	CTAACCGTATCTGGACACATCCGAGTGACCGTG
K36I	CTAACCGTATCTGGAATCATCCGAGTGACCGTG

K36L	CTAACCGTATCTGGACTGATCCGAGTGACCGTG
K36M	CTAACCGTATCTGGAATGATCCGAGTGACCGTG
K36F	CTAACCGTATCTGGATTCATCCGAGTGACCGTG
K36P	CTAACCGTATCTGGACCCATCCGAGTGACCGTG
K36S	CTAACCGTATCTGGAAGCATCCGAGTGACCGTG
K36T	CTAACCGTATCTGGAACCATCCGAGTGACCGTG
K36W	CTAACCGTATCTGGATGGATCCGAGTGACCGTG
K36Y	CTAACCGTATCTGGATACATCCGAGTGACCGTG
K36V	CTAACCGTATCTGGAGTGATCCGAGTGACCGTG
T40A	GGAAAGATCCGAGTGGCCGTGACTTTCTTCCTT
T40R	GGAAAGATCCGAGTGAGAGTGACTTTCTTCCTT
T40N	GGAAAGATCCGAGTGAACGTGACTTTCTTCCTT
T40D	GGAAAGATCCGAGTGGACGTGACTTTCTTCCTT
T40C	GGAAAGATCCGAGTGTGCGTGACTTTCTTCCTT
T40Q	GGAAAGATCCGAGTGCAGGTGACTTTCTTCCTT
T40E	GGAAAGATCCGAGTGGAGGTGACTTTCTTCCTT
T40G	GGAAAGATCCGAGTGGGCGTGACTTTCTTCCTT
T40H	GGAAAGATCCGAGTGCACGTGACTTTCTTCCTT
T40I	GGAAAGATCCGAGTGATCGTGACTTTCTTCCTT
T40L	GGAAAGATCCGAGTGCTGGTGACTTTCTTCCTT
T40K	GGAAAGATCCGAGTGAAGGTGACTTTCTTCCTT
T40M	GGAAAGATCCGAGTGATGGTGACTTTCTTCCTT
T40F	GGAAAGATCCGAGTGTTCGTGACTTTCTTCCTT
T40P	GGAAAGATCCGAGTGCCCGTGACTTTCTTCCTT
T40S	GGAAAGATCCGAGTGAGCGTGACTTTCTTCCTT
T40W	GGAAAGATCCGAGTGTGGGTGACTTTCTTCCTT
T40Y	GGAAAGATCCGAGTGTACGTGACTTTCTTCCTT
T40V	GGAAAGATCCGAGTGGTGGTGACTTTCTTCCTT
T42A	ATCCGAGTGACCGTGGCCTTCTTCCTTTTCCTA
T42R	ATCCGAGTGACCGTGAGATTCTTCCTTTTCCTA
T42N	ATCCGAGTGACCGTGAACTTCTTCCTTTTCCTA
T42D	ATCCGAGTGACCGTGGACTTCTTCCTTTTCCTA
T42C	ATCCGAGTGACCGTGTGCTTCTTCCTTTTCCTA
T42Q	ATCCGAGTGACCGTGCAGTTCTTCCTTTTCCTA
T42E	ATCCGAGTGACCGTGGAGTTCTTCCTTTTCCTA
T42G	ATCCGAGTGACCGTGGGCTTCTTCCTTTTCCTA
T42H	ATCCGAGTGACCGTGCACTTCTTCCTTTTCCTA
T42I	ATCCGAGTGACCGTGATCTTCTTCCTTTTCCTA
T42L	ATCCGAGTGACCGTGCTGTTCTTCCTTTTCCTA
T42K	ATCCGAGTGACCGTGAAGTTCTTCCTTTTCCTA
T42M	ATCCGAGTGACCGTGATGTTCTTCCTTTTCCTA

T42F	ATCCGAGTGACCGTGTTCTTCTTCCTTTTCCTA
T42P	ATCCGAGTGACCGTGCCCTTCTTCCTTTTCCTA
T42S	ATCCGAGTGACCGTGAGCTTCTTCCTTTTCCTA
T42W	ATCCGAGTGACCGTGTGGTTCTTCCTTTTCCTA
T42Y	ATCCGAGTGACCGTGTACTTCTTCCTTTTCCTA
T42V	ATCCGAGTGACCGTGGTGTTCTTCCTTTTCCTA
F44A	GTGACCGTGACTTTCGCCCTTTTCCTACTCTCT
F44R	GTGACCGTGACTTTCAGACTTTTCCTACTCTCT
F44N	GTGACCGTGACTTTCAACCTTTTCCTACTCTCT
F44D	GTGACCGTGACTTTCGACCTTTTCCTACTCTCT
F44C	GTGACCGTGACTTTCTGCCTTTTCCTACTCTCT
F44Q	GTGACCGTGACTTTCCAGCTTTTCCTACTCTCT
F44E	GTGACCGTGACTTTCGAGCTTTTCCTACTCTCT
F44G	GTGACCGTGACTTTCGGCCTTTTCCTACTCTCT
F44H	GTGACCGTGACTTTCCACCTTTTCCTACTCTCT
F44I	GTGACCGTGACTTTCATCCTTTTCCTACTCTCT
F44L	GTGACCGTGACTTTCCTGCTTTTCCTACTCTCT
F44K	GTGACCGTGACTTTCAAGCTTTTCCTACTCTCT
F44M	GTGACCGTGACTTTCATGCTTTTCCTACTCTCT
F44P	GTGACCGTGACTTTCCCCCTTTTCCTACTCTCT
F44S	GTGACCGTGACTTTCAGCCTTTTCCTACTCTCT
F44T	GTGACCGTGACTTTCACCCTTTTCCTACTCTCT
F44W	GTGACCGTGACTTTCTGGCTTTTCCTACTCTCT
F44Y	GTGACCGTGACTTTCTACCTTTTCCTACTCTCT
F44V	GTGACCGTGACTTTCGTGCTTTTCCTACTCTCT
S49A	TTCCTTTTCCTACTCGCCACTGCCTTCAATGCT
S49R	TTCCTTTTCCTACTCAGAACTGCCTTCAATGCT
S49N	TTCCTTTTCCTACTCAACACTGCCTTCAATGCT
S49D	TTCCTTTTCCTACTCGACACTGCCTTCAATGCT
S49C	TTCCTTTTCCTACTCTGCACTGCCTTCAATGCT
S49Q	TTCCTTTTCCTACTCCAGACTGCCTTCAATGCT
S49E	TTCCTTTTCCTACTCGAGACTGCCTTCAATGCT
S49G	TTCCTTTTCCTACTCGGCACTGCCTTCAATGCT
S49H	TTCCTTTTCCTACTCCACACTGCCTTCAATGCT
S49I	TTCCTTTTCCTACTCATCACTGCCTTCAATGCT
S49L	TTCCTTTTCCTACTCCTGACTGCCTTCAATGCT
S49K	TTCCTTTTCCTACTCAAGACTGCCTTCAATGCT
S49M	TTCCTTTTCCTACTCATGACTGCCTTCAATGCT
S49F	TTCCTTTTCCTACTCTTCACTGCCTTCAATGCT
S49P	TTCCTTTTCCTACTCCCCACTGCCTTCAATGCT
S49T	TTCCTTTTCCTACTCACCACTGCCTTCAATGCT

S49W	TTCCTTTTCCTACTCTGGACTGCCTTCAATGCT
S49Y	TTCCTTTTCCTACTCTACACTGCCTTCAATGCT
S49V	TTCCTTTTCCTACTCGTGACTGCCTTCAATGCT
A51R	TTCCTACTCTCTACTAGATTCAATGCTTCCTTC
A51N	TTCCTACTCTCTACTAACTTCAATGCTTCCTTC
A51D	TTCCTACTCTCTACTGACTTCAATGCTTCCTTC
A51C	TTCCTACTCTCTACTTGCTTCAATGCTTCCTTC
A51Q	TTCCTACTCTCTACTCAGTTCAATGCTTCCTTC
A51E	TTCCTACTCTCTACTGAGTTCAATGCTTCCTTC
A51G	TTCCTACTCTCTACTGGCTTCAATGCTTCCTTC
A51H	TTCCTACTCTCTACTCACTTCAATGCTTCCTTC
A51I	TTCCTACTCTCTACTATCTTCAATGCTTCCTTC
A51L	TTCCTACTCTCTACTCTGTTCAATGCTTCCTTC
A51K	TTCCTACTCTCTACTAAGTTCAATGCTTCCTTC
A51M	TTCCTACTCTCTACTATGTTCAATGCTTCCTTC
A51F	TTCCTACTCTCTACTTTCTTCAATGCTTCCTTC
A51P	TTCCTACTCTCTACTCCCTTCAATGCTTCCTTC
A51S	TTCCTACTCTCTACTAGCTTCAATGCTTCCTTC
A51T	TTCCTACTCTCTACTACCTTCAATGCTTCCTTC
A51W	TTCCTACTCTCTACTTGGTTCAATGCTTCCTTC
A51Y	TTCCTACTCTCTACTTACTTCAATGCTTCCTTC
A51V	TTCCTACTCTCTACTGTGTTCAATGCTTCCTTC
N53A	CTCTCTACTGCCTTCGCCGCTTCCTTCTTGTTG
N53R	CTCTCTACTGCCTTCAGAGCTTCCTTCTTGTTG
N53D	CTCTCTACTGCCTTCGACGCTTCCTTCTTGTTG
N53C	CTCTCTACTGCCTTCTGCGCTTCCTTCTTGTTG
N53Q	CTCTCTACTGCCTTCCAGGCTTCCTTCTTGTTG
N53E	CTCTCTACTGCCTTCGAGGCTTCCTTCTTGTTG
N53G	CTCTCTACTGCCTTCGGCGCTTCCTTCTTGTTG
N53H	CTCTCTACTGCCTTCCACGCTTCCTTCTTGTTG
N53I	CTCTCTACTGCCTTCATCGCTTCCTTCTTGTTG
N53L	CTCTCTACTGCCTTCCTGGCTTCCTTCTTGTTG
N53K	CTCTCTACTGCCTTCAAGGCTTCCTTCTTGTTG
N53M	CTCTCTACTGCCTTCATGGCTTCCTTCTTGTTG
N53F	CTCTCTACTGCCTTCTTCGCTTCCTTCTTGTTG
N53P	CTCTCTACTGCCTTCCCCGCTTCCTTCTTGTTG
N53S	CTCTCTACTGCCTTCAGCGCTTCCTTCTTGTTG
N53T	CTCTCTACTGCCTTCACCGCTTCCTTCTTGTTG
N53W	CTCTCTACTGCCTTCTGGGCTTCCTTCTTGTTG
N53Y	CTCTCTACTGCCTTCTACGCTTCCTTCTTGTTG
N53V	CTCTCTACTGCCTTCGTGGCTTCCTTCTTGTTG

T64A	AAGCTGCAGAAGTGGGCCCAGAAGAGGAAGAAA
T64R	AAGCTGCAGAAGTGGAGACAGAAGAGGAAGAAA
T64N	AAGCTGCAGAAGTGGAACCAGAAGAGGAAGAAA
T64D	AAGCTGCAGAAGTGGGACCAGAAGAGGAAGAAA
T64C	AAGCTGCAGAAGTGGTGCCAGAAGAGGAAGAAA
T64Q	AAGCTGCAGAAGTGGCAGCAGAAGAGGAAGAAA
T64E	AAGCTGCAGAAGTGGGAGCAGAAGAGGAAGAAA
T64G	AAGCTGCAGAAGTGGGGCCAGAAGAGGAAGAAA
T64H	AAGCTGCAGAAGTGGCACCAGAAGAGGAAGAAA
T64I	AAGCTGCAGAAGTGGATCCAGAAGAGGAAGAAA
T64L	AAGCTGCAGAAGTGGCTGCAGAAGAGGAAGAAA
T64K	AAGCTGCAGAAGTGGAAGCAGAAGAGGAAGAAA
T64M	AAGCTGCAGAAGTGGATGCAGAAGAGGAAGAAA
T64F	AAGCTGCAGAAGTGGTTCCAGAAGAGGAAGAAA
T64P	AAGCTGCAGAAGTGGCCCCAGAAGAGGAAGAAA
T64S	AAGCTGCAGAAGTGGAGCCAGAAGAGGAAGAAA
T64W	AAGCTGCAGAAGTGGTGGCAGAAGAGGAAGAAA
T64Y	AAGCTGCAGAAGTGGTACCAGAAGAGGAAGAAA
T64V	AAGCTGCAGAAGTGGGTGCAGAAGAGGAAGAAA
K68A	TGGACTCAGAAGAGGGCCAAAGGAAAAAAGCTC
K68R	TGGACTCAGAAGAGGAGAAAAGGAAAAAAGCTC
K68N	TGGACTCAGAAGAGGAACAAAGGAAAAAAGCTC
K68D	TGGACTCAGAAGAGGGACAAAGGAAAAAAGCTC
K68C	TGGACTCAGAAGAGGTGCAAAGGAAAAAAGCTC
K68Q	TGGACTCAGAAGAGGCAGAAAGGAAAAAAGCTC
K68E	TGGACTCAGAAGAGGGAGAAAGGAAAAAAGCTC
K68G	TGGACTCAGAAGAGGGGCAAAGGAAAAAAGCTC
K68H	TGGACTCAGAAGAGGCACAAAGGAAAAAAGCTC
K68I	TGGACTCAGAAGAGGATCAAAGGAAAAAAGCTC
K68L	TGGACTCAGAAGAGGCTGAAAGGAAAAAAGCTC
K68M	TGGACTCAGAAGAGGATGAAAGGAAAAAAGCTC
K68F	TGGACTCAGAAGAGGTTCAAAGGAAAAAAGCTC
K68P	TGGACTCAGAAGAGGCCCAAAGGAAAAAAGCTC
K68S	TGGACTCAGAAGAGGAGCAAAGGAAAAAAGCTC
K68T	TGGACTCAGAAGAGGACCAAAGGAAAAAAGCTC
K68W	TGGACTCAGAAGAGGTGGAAAGGAAAAAAGCTC
K68Y	TGGACTCAGAAGAGGTACAAAGGAAAAAAGCTC
K68V	TGGACTCAGAAGAGGGTGAAAGGAAAAAAGCTC
K72A	AGGAAGAAAGGAAAAGCCCTCTCAAGGATGAAG
K72R	AGGAAGAAAGGAAAAAGACTCTCAAGGATGAAG
K72N	AGGAAGAAAGGAAAAAACCTCTCAAGGATGAAG

K72D	AGGAAGAAAGGAAAAGACCTCTCAAGGATGAAG
K72C	AGGAAGAAAGGAAAATGCCTCTCAAGGATGAAG
K72Q	AGGAAGAAAGGAAAACAGCTCTCAAGGATGAAG
K72E	AGGAAGAAAGGAAAAGAGCTCTCAAGGATGAAG
K72G	AGGAAGAAAGGAAAAGGCCTCTCAAGGATGAAG
K72H	AGGAAGAAAGGAAAACACCTCTCAAGGATGAAG
K72I	AGGAAGAAAGGAAAAATCCTCTCAAGGATGAAG
K72L	AGGAAGAAAGGAAAACTGCTCTCAAGGATGAAG
K72M	AGGAAGAAAGGAAAAATGCTCTCAAGGATGAAG
K72F	AGGAAGAAAGGAAAATTCCTCTCAAGGATGAAG
K72P	AGGAAGAAAGGAAAACCCCTCTCAAGGATGAAG
K72S	AGGAAGAAAGGAAAAAGCCTCTCAAGGATGAAG
K72T	AGGAAGAAAGGAAAAACCCTCTCAAGGATGAAG
K72W	AGGAAGAAAGGAAAATGGCTCTCAAGGATGAAG
K72Y	AGGAAGAAAGGAAAATACCTCTCAAGGATGAAG
K72V	AGGAAGAAAGGAAAAGTGCTCTCAAGGATGAAG
M76A	AAAAAGCTCTCAAGGGCCAAGGTGCTTTTAAAG
M76R	AAAAAGCTCTCAAGGAGAAAGGTGCTTTTAAAG
M76N	AAAAAGCTCTCAAGGAACAAGGTGCTTTTAAAG
M76D	AAAAAGCTCTCAAGGGACAAGGTGCTTTTAAAG
M76C	AAAAAGCTCTCAAGGTGCAAGGTGCTTTTAAAG
M76Q	AAAAAGCTCTCAAGGCAGAAGGTGCTTTTAAAG
M76E	AAAAAGCTCTCAAGGGAGAAGGTGCTTTTAAAG
M76G	AAAAAGCTCTCAAGGGGCAAGGTGCTTTTAAAG
M76H	AAAAAGCTCTCAAGGCACAAGGTGCTTTTAAAG
M76I	AAAAAGCTCTCAAGGATCAAGGTGCTTTTAAAG
M76L	AAAAAGCTCTCAAGGCTGAAGGTGCTTTTAAAG
M76K	AAAAAGCTCTCAAGGAAGAAGGTGCTTTTAAAG
M76F	AAAAAGCTCTCAAGGTTCAAGGTGCTTTTAAAG
M76P	AAAAAGCTCTCAAGGCCCAAGGTGCTTTTAAAG
M76S	AAAAAGCTCTCAAGGAGCAAGGTGCTTTTAAAG
M76T	AAAAAGCTCTCAAGGACCAAGGTGCTTTTAAAG
M76W	AAAAAGCTCTCAAGGTGGAAGGTGCTTTTAAAG
M76Y	AAAAAGCTCTCAAGGTACAAGGTGCTTTTAAAG
M76V	AAAAAGCTCTCAAGGGTGAAGGTGCTTTTAAAG
T84A	CTTTTAAAGCATTTGGCCTTAGCCAACCTGCTG
T84R	CTTTTAAAGCATTTGAGATTAGCCAACCTGCTG
T84N	CTTTTAAAGCATTTGAACTTAGCCAACCTGCTG
T84D	CTTTTAAAGCATTTGGACTTAGCCAACCTGCTG
T84C	CTTTTAAAGCATTTGTGCTTAGCCAACCTGCTG
T84Q	CTTTTAAAGCATTTGCAGTTAGCCAACCTGCTG

T84E	CTTTTAAAGCATTTGGAGTTAGCCAACCTGCTG
T84G	CTTTTAAAGCATTTGGGCTTAGCCAACCTGCTG
T84H	CTTTTAAAGCATTTGCACTTAGCCAACCTGCTG
T84I	CTTTTAAAGCATTTGATCTTAGCCAACCTGCTG
T84L	CTTTTAAAGCATTTGCTGTTAGCCAACCTGCTG
T84K	CTTTTAAAGCATTTGAAGTTAGCCAACCTGCTG
T84M	CTTTTAAAGCATTTGATGTTAGCCAACCTGCTG
T84F	CTTTTAAAGCATTTGTTCTTAGCCAACCTGCTG
T84P	CTTTTAAAGCATTTGCCCTTAGCCAACCTGCTG
T84S	CTTTTAAAGCATTTGAGCTTAGCCAACCTGCTG
T84W	CTTTTAAAGCATTTGTGGTTAGCCAACCTGCTG
T84Y	CTTTTAAAGCATTTGTACTTAGCCAACCTGCTG
T84V	CTTTTAAAGCATTTGGTGTTAGCCAACCTGCTG
L85A	TTAAAGCATTTGACCGCCGCCAACCTGCTGGAG
L85R	TTAAAGCATTTGACCAGAGCCAACCTGCTGGAG
L85N	TTAAAGCATTTGACCAACGCCAACCTGCTGGAG
L85D	TTAAAGCATTTGACCGACGCCAACCTGCTGGAG
L85C	TTAAAGCATTTGACCTGCGCCAACCTGCTGGAG
L85Q	TTAAAGCATTTGACCCAGGCCAACCTGCTGGAG
L85E	TTAAAGCATTTGACCGAGGCCAACCTGCTGGAG
L85G	TTAAAGCATTTGACCGGCGCCAACCTGCTGGAG
L85H	TTAAAGCATTTGACCCACGCCAACCTGCTGGAG
L85I	TTAAAGCATTTGACCATCGCCAACCTGCTGGAG
L85K	TTAAAGCATTTGACCAAGGCCAACCTGCTGGAG
L85M	TTAAAGCATTTGACCATGGCCAACCTGCTGGAG
L85F	TTAAAGCATTTGACCTTCGCCAACCTGCTGGAG
L85P	TTAAAGCATTTGACCCCCGCCAACCTGCTGGAG
L85S	TTAAAGCATTTGACCAGCGCCAACCTGCTGGAG
L85T	TTAAAGCATTTGACCACCGCCAACCTGCTGGAG
L85W	TTAAAGCATTTGACCTGGGCCAACCTGCTGGAG
L85Y	TTAAAGCATTTGACCTACGCCAACCTGCTGGAG
L85V	TTAAAGCATTTGACCGTGGCCAACCTGCTGGAG
L89A	ACCTTAGCCAACCTGGCCGAGACTCTGATCGTC
L89R	ACCTTAGCCAACCTGAGAGAGACTCTGATCGTC
L89N	ACCTTAGCCAACCTGAACGAGACTCTGATCGTC
L89D	ACCTTAGCCAACCTGGACGAGACTCTGATCGTC
L89C	ACCTTAGCCAACCTGTGCGAGACTCTGATCGTC
L89Q	ACCTTAGCCAACCTGCAGGAGACTCTGATCGTC
L89E	ACCTTAGCCAACCTGGAGGAGACTCTGATCGTC
L89G	ACCTTAGCCAACCTGGGCGAGACTCTGATCGTC
L89H	ACCTTAGCCAACCTGCACGAGACTCTGATCGTC

L89I	ACCTTAGCCAACCTGATCGAGACTCTGATCGTC
L89K	ACCTTAGCCAACCTGAAGGAGACTCTGATCGTC
L89M	ACCTTAGCCAACCTGATGGAGACTCTGATCGTC
L89F	ACCTTAGCCAACCTGTTCGAGACTCTGATCGTC
L89P	ACCTTAGCCAACCTGCCCGAGACTCTGATCGTC
L89S	ACCTTAGCCAACCTGAGCGAGACTCTGATCGTC
L89T	ACCTTAGCCAACCTGACCGAGACTCTGATCGTC
L89W	ACCTTAGCCAACCTGTGGGAGACTCTGATCGTC
L89Y	ACCTTAGCCAACCTGTACGAGACTCTGATCGTC
L89V	ACCTTAGCCAACCTGGTGGAGACTCTGATCGTC
L92A	AACCTGCTGGAGACTGCCATCGTCATGCCACTG
L92R	AACCTGCTGGAGACTAGAATCGTCATGCCACTG
L92N	AACCTGCTGGAGACTAACATCGTCATGCCACTG
L92D	AACCTGCTGGAGACTGACATCGTCATGCCACTG
L92C	AACCTGCTGGAGACTTGCATCGTCATGCCACTG
L92Q	AACCTGCTGGAGACTCAGATCGTCATGCCACTG
L92E	AACCTGCTGGAGACTGAGATCGTCATGCCACTG
L92G	AACCTGCTGGAGACTGGCATCGTCATGCCACTG
L92H	AACCTGCTGGAGACTCACATCGTCATGCCACTG
L92I	AACCTGCTGGAGACTATCATCGTCATGCCACTG
L92K	AACCTGCTGGAGACTAAGATCGTCATGCCACTG
L92M	AACCTGCTGGAGACTATGATCGTCATGCCACTG
L92F	AACCTGCTGGAGACTTTCATCGTCATGCCACTG
L92P	AACCTGCTGGAGACTCCCATCGTCATGCCACTG
L92S	AACCTGCTGGAGACTAGCATCGTCATGCCACTG
L92T	AACCTGCTGGAGACTACCATCGTCATGCCACTG
L92W	AACCTGCTGGAGACTTGGATCGTCATGCCACTG
L92Y	AACCTGCTGGAGACTTACATCGTCATGCCACTG
L92V	AACCTGCTGGAGACTGTGATCGTCATGCCACTG
M95A	GAGACTCTGATCGTCGCCCCACTGGATGGGATG
M95R	GAGACTCTGATCGTCAGACCACTGGATGGGATG
M95N	GAGACTCTGATCGTCAACCCACTGGATGGGATG
M95D	GAGACTCTGATCGTCGACCCACTGGATGGGATG
M95C	GAGACTCTGATCGTCTGCCCACTGGATGGGATG
M95Q	GAGACTCTGATCGTCCAGCCACTGGATGGGATG
M95E	GAGACTCTGATCGTCGAGCCACTGGATGGGATG
M95G	GAGACTCTGATCGTCGGCCCACTGGATGGGATG
M95H	GAGACTCTGATCGTCCACCCACTGGATGGGATG
M95I	GAGACTCTGATCGTCATCCCACTGGATGGGATG
M95L	GAGACTCTGATCGTCCTGCCACTGGATGGGATG
M95K	GAGACTCTGATCGTCAAGCCACTGGATGGGATG

M95F	GAGACTCTGATCGTCTTCCCACTGGATGGGATG
M95P	GAGACTCTGATCGTCCCCCCACTGGATGGGATG
M95S	GAGACTCTGATCGTCAGCCCACTGGATGGGATG
M95T	GAGACTCTGATCGTCACCCCACTGGATGGGATG
M95W	GAGACTCTGATCGTCTGGCCACTGGATGGGATG
M95Y	GAGACTCTGATCGTCTACCCACTGGATGGGATG
M95V	GAGACTCTGATCGTCGTGCCACTGGATGGGATG
D98A	ATCGTCATGCCACTGGCCGGGATGTGGAATATT
D98R	ATCGTCATGCCACTGAGAGGGATGTGGAATATT
D98N	ATCGTCATGCCACTGAACGGGATGTGGAATATT
D98C	ATCGTCATGCCACTGTGCGGGATGTGGAATATT
D98Q	ATCGTCATGCCACTGCAGGGGATGTGGAATATT
D98E	ATCGTCATGCCACTGGAGGGGATGTGGAATATT
D98G	ATCGTCATGCCACTGGGCGGGATGTGGAATATT
D98H	ATCGTCATGCCACTGCACGGGATGTGGAATATT
D98I	ATCGTCATGCCACTGATCGGGATGTGGAATATT
D98L	ATCGTCATGCCACTGCTGGGGATGTGGAATATT
D98K	ATCGTCATGCCACTGAAGGGGATGTGGAATATT
D98M	ATCGTCATGCCACTGATGGGGATGTGGAATATT
D98F	ATCGTCATGCCACTGTTCGGGATGTGGAATATT
D98P	ATCGTCATGCCACTGCCCGGGATGTGGAATATT
D98S	ATCGTCATGCCACTGAGCGGGATGTGGAATATT
D98T	ATCGTCATGCCACTGACCGGGATGTGGAATATT
D98W	ATCGTCATGCCACTGTGGGGGATGTGGAATATT
D98Y	ATCGTCATGCCACTGTACGGGATGTGGAATATT
D98V	ATCGTCATGCCACTGGTGGGGATGTGGAATATT
V105A	ATGTGGAATATTACTGCCCAGTGGTATGCTGGG
V105R	ATGTGGAATATTACTAGACAGTGGTATGCTGGG
V105N	ATGTGGAATATTACTAACCAGTGGTATGCTGGG
V105D	ATGTGGAATATTACTGACCAGTGGTATGCTGGG
V105C	ATGTGGAATATTACTTGCCAGTGGTATGCTGGG
V105Q	ATGTGGAATATTACTCAGCAGTGGTATGCTGGG
V105E	ATGTGGAATATTACTGAGCAGTGGTATGCTGGG
V105G	ATGTGGAATATTACTGGCCAGTGGTATGCTGGG
V105H	ATGTGGAATATTACTCACCAGTGGTATGCTGGG
V105I	ATGTGGAATATTACTATCCAGTGGTATGCTGGG
V105L	ATGTGGAATATTACTCTGCAGTGGTATGCTGGG
V105K	ATGTGGAATATTACTAAGCAGTGGTATGCTGGG
V105M	ATGTGGAATATTACTATGCAGTGGTATGCTGGG
V105F	ATGTGGAATATTACTTTCCAGTGGTATGCTGGG
V105P	ATGTGGAATATTACTCCCCAGTGGTATGCTGGG

V105S	ATGTGGAATATTACTAGCCAGTGGTATGCTGGG
V105T	ATGTGGAATATTACTACCCAGTGGTATGCTGGG
V105W	ATGTGGAATATTACTTGGCAGTGGTATGCTGGG
V105Y	ATGTGGAATATTACTTACCAGTGGTATGCTGGG
Q106A	TGGAATATTACTGTTGCCTGGTATGCTGGGGAG
Q106R	TGGAATATTACTGTTAGATGGTATGCTGGGGAG
Q106N	TGGAATATTACTGTTAACTGGTATGCTGGGGAG
Q106D	TGGAATATTACTGTTGACTGGTATGCTGGGGAG
Q106C	TGGAATATTACTGTTTGCTGGTATGCTGGGGAG
Q106E	TGGAATATTACTGTTGAGTGGTATGCTGGGGAG
Q106G	TGGAATATTACTGTTGGCTGGTATGCTGGGGAG
Q106H	TGGAATATTACTGTTCACTGGTATGCTGGGGAG
Q106I	TGGAATATTACTGTTATCTGGTATGCTGGGGAG
Q106L	TGGAATATTACTGTTCTGTGGTATGCTGGGGAG
Q106K	TGGAATATTACTGTTAAGTGGTATGCTGGGGAG
Q106M	TGGAATATTACTGTTATGTGGTATGCTGGGGAG
Q106F	TGGAATATTACTGTTTTCTGGTATGCTGGGGAG
Q106P	TGGAATATTACTGTTCCCTGGTATGCTGGGGAG
Q106S	TGGAATATTACTGTTAGCTGGTATGCTGGGGAG
Q106T	TGGAATATTACTGTTACCTGGTATGCTGGGGAG
Q106W	TGGAATATTACTGTTTGGTGGTATGCTGGGGAG
Q106Y	TGGAATATTACTGTTTACTGGTATGCTGGGGAG
Q106V	TGGAATATTACTGTTGTGTGGTATGCTGGGGAG
Y108A	ATTACTGTTCAGTGGGCCGCTGGGGAGTTCCTC
Y108R	ATTACTGTTCAGTGGAGAGCTGGGGAGTTCCTC
Y108N	ATTACTGTTCAGTGGAACGCTGGGGAGTTCCTC
Y108D	ATTACTGTTCAGTGGGACGCTGGGGAGTTCCTC
Y108C	ATTACTGTTCAGTGGTGCGCTGGGGAGTTCCTC
Y108Q	ATTACTGTTCAGTGGCAGGCTGGGGAGTTCCTC
Y108E	ATTACTGTTCAGTGGGAGGCTGGGGAGTTCCTC
Y108G	ATTACTGTTCAGTGGGGCGCTGGGGAGTTCCTC
Y108H	ATTACTGTTCAGTGGCACGCTGGGGAGTTCCTC
Y108I	ATTACTGTTCAGTGGATCGCTGGGGAGTTCCTC
Y108L	ATTACTGTTCAGTGGCTGGCTGGGGAGTTCCTC
Y108K	ATTACTGTTCAGTGGAAGGCTGGGGAGTTCCTC
Y108M	ATTACTGTTCAGTGGATGGCTGGGGAGTTCCTC
Y108F	ATTACTGTTCAGTGGTTCGCTGGGGAGTTCCTC
Y108P	ATTACTGTTCAGTGGCCCGCTGGGGAGTTCCTC
Y108S	ATTACTGTTCAGTGGAGCGCTGGGGAGTTCCTC
Y108T	ATTACTGTTCAGTGGACCGCTGGGGAGTTCCTC
Y108W	ATTACTGTTCAGTGGTGGGCTGGGGAGTTCCTC

Y108V	ATTACTGTTCAGTGGGTGGCTGGGGAGTTCCTC
A109R	ACTGTTCAGTGGTATAGAGGGGAGTTCCTCTGC
A109N	ACTGTTCAGTGGTATAACGGGGAGTTCCTCTGC
A109D	ACTGTTCAGTGGTATGACGGGGAGTTCCTCTGC
A109C	ACTGTTCAGTGGTATTGCGGGGAGTTCCTCTGC
A109Q	ACTGTTCAGTGGTATCAGGGGGAGTTCCTCTGC
A109E	ACTGTTCAGTGGTATGAGGGGGAGTTCCTCTGC
A109G	ACTGTTCAGTGGTATGGCGGGGAGTTCCTCTGC
A109H	ACTGTTCAGTGGTATCACGGGGAGTTCCTCTGC
A109I	ACTGTTCAGTGGTATATCGGGGAGTTCCTCTGC
A109L	ACTGTTCAGTGGTATCTGGGGGAGTTCCTCTGC
A109K	ACTGTTCAGTGGTATAAGGGGGAGTTCCTCTGC
A109M	ACTGTTCAGTGGTATATGGGGGAGTTCCTCTGC
A109F	ACTGTTCAGTGGTATTTCGGGGAGTTCCTCTGC
A109P	ACTGTTCAGTGGTATCCCGGGGAGTTCCTCTGC
A109S	ACTGTTCAGTGGTATAGCGGGGAGTTCCTCTGC
A109T	ACTGTTCAGTGGTATACCGGGGAGTTCCTCTGC
A109W	ACTGTTCAGTGGTATTGGGGGGAGTTCCTCTGC
A109Y	ACTGTTCAGTGGTATTACGGGGAGTTCCTCTGC
A109V	ACTGTTCAGTGGTATGTGGGGGAGTTCCTCTGC
F112A	TGGTATGCTGGGGAGGCCCTCTGCAAAGTTCTC
F112R	TGGTATGCTGGGGAGAGACTCTGCAAAGTTCTC
F112N	TGGTATGCTGGGGAGAACCTCTGCAAAGTTCTC
F112D	TGGTATGCTGGGGAGGACCTCTGCAAAGTTCTC
F112C	TGGTATGCTGGGGAGTGCCTCTGCAAAGTTCTC
F112Q	TGGTATGCTGGGGAGCAGCTCTGCAAAGTTCTC
F112E	TGGTATGCTGGGGAGGAGCTCTGCAAAGTTCTC
F112G	TGGTATGCTGGGGAGGGCCTCTGCAAAGTTCTC
F112H	TGGTATGCTGGGGAGCACCTCTGCAAAGTTCTC
F112I	TGGTATGCTGGGGAGATCCTCTGCAAAGTTCTC
F112L	TGGTATGCTGGGGAGCTGCTCTGCAAAGTTCTC
F112K	TGGTATGCTGGGGAGAAGCTCTGCAAAGTTCTC
F112M	TGGTATGCTGGGGAGATGCTCTGCAAAGTTCTC
F112P	TGGTATGCTGGGGAGCCCCTCTGCAAAGTTCTC
F112S	TGGTATGCTGGGGAGAGCCTCTGCAAAGTTCTC
F112T	TGGTATGCTGGGGAGACCCTCTGCAAAGTTCTC
F112W	TGGTATGCTGGGGAGTGGCTCTGCAAAGTTCTC
F112Y	TGGTATGCTGGGGAGTACCTCTGCAAAGTTCTC
F112V	TGGTATGCTGGGGAGGTGCTCTGCAAAGTTCTC
V116A	GAGTTCCTCTGCAAAGCCCTCAGCTATCTGAAG
V116R	GAGTTCCTCTGCAAAAGACTCAGCTATCTGAAG

V116N	GAGTTCCTCTGCAAAAACCTCAGCTATCTGAAG
V116D	GAGTTCCTCTGCAAAGACCTCAGCTATCTGAAG
V116C	GAGTTCCTCTGCAAATGCCTCAGCTATCTGAAG
V116Q	GAGTTCCTCTGCAAACAGCTCAGCTATCTGAAG
V116E	GAGTTCCTCTGCAAAGAGCTCAGCTATCTGAAG
V116G	GAGTTCCTCTGCAAAGGCCTCAGCTATCTGAAG
V116H	GAGTTCCTCTGCAAACACCTCAGCTATCTGAAG
V116I	GAGTTCCTCTGCAAAATCCTCAGCTATCTGAAG
V116L	GAGTTCCTCTGCAAACTGCTCAGCTATCTGAAG
V116K	GAGTTCCTCTGCAAAAAGCTCAGCTATCTGAAG
V116M	GAGTTCCTCTGCAAAATGCTCAGCTATCTGAAG
V116F	GAGTTCCTCTGCAAATTCCTCAGCTATCTGAAG
V116P	GAGTTCCTCTGCAAACCCCTCAGCTATCTGAAG
V116S	GAGTTCCTCTGCAAAAGCCTCAGCTATCTGAAG
V116T	GAGTTCCTCTGCAAAACCCTCAGCTATCTGAAG
V116W	GAGTTCCTCTGCAAATGGCTCAGCTATCTGAAG
V116Y	GAGTTCCTCTGCAAATACCTCAGCTATCTGAAG
L120A	AAAGTTCTCAGCTATGCCAAGCTCTTCTCTATG
L120R	AAAGTTCTCAGCTATAGAAAGCTCTTCTCTATG
L120N	AAAGTTCTCAGCTATAACAAGCTCTTCTCTATG
L120D	AAAGTTCTCAGCTATGACAAGCTCTTCTCTATG
L120C	AAAGTTCTCAGCTATTGCAAGCTCTTCTCTATG
L120Q	AAAGTTCTCAGCTATCAGAAGCTCTTCTCTATG
L120E	AAAGTTCTCAGCTATGAGAAGCTCTTCTCTATG
L120G	AAAGTTCTCAGCTATGGCAAGCTCTTCTCTATG
L120H	AAAGTTCTCAGCTATCACAAGCTCTTCTCTATG
L120I	AAAGTTCTCAGCTATATCAAGCTCTTCTCTATG
L120K	AAAGTTCTCAGCTATAAGAAGCTCTTCTCTATG
L120M	AAAGTTCTCAGCTATATGAAGCTCTTCTCTATG
L120F	AAAGTTCTCAGCTATTTCAAGCTCTTCTCTATG
L120P	AAAGTTCTCAGCTATCCCAAGCTCTTCTCTATG
L120S	AAAGTTCTCAGCTATAGCAAGCTCTTCTCTATG
L120T	AAAGTTCTCAGCTATACCAAGCTCTTCTCTATG
L120W	AAAGTTCTCAGCTATTGGAAGCTCTTCTCTATG
L120Y	AAAGTTCTCAGCTATTACAAGCTCTTCTCTATG
L120V	AAAGTTCTCAGCTATGTGAAGCTCTTCTCTATG
A129R	TCTATGTATGCCCCAAGATTCATGATGGTGGTG
A129N	TCTATGTATGCCCCAAACTTCATGATGGTGGTG
A129D	TCTATGTATGCCCCAGACTTCATGATGGTGGTG
A129C	TCTATGTATGCCCCATGCTTCATGATGGTGGTG
A129Q	TCTATGTATGCCCCACAGTTCATGATGGTGGTG

A129E	TCTATGTATGCCCCAGAGTTCATGATGGTGGTG
A129G	TCTATGTATGCCCCAGGCTTCATGATGGTGGTG
A129H	TCTATGTATGCCCCACACTTCATGATGGTGGTG
A129I	TCTATGTATGCCCCAATCTTCATGATGGTGGTG
A129L	TCTATGTATGCCCCACTGTTCATGATGGTGGTG
A129K	TCTATGTATGCCCCAAAGTTCATGATGGTGGTG
A129M	TCTATGTATGCCCCAATGTTCATGATGGTGGTG
A129F	TCTATGTATGCCCCATTCTTCATGATGGTGGTG
A129P	TCTATGTATGCCCCACCCTTCATGATGGTGGTG
A129S	TCTATGTATGCCCCAAGCTTCATGATGGTGGTG
A129T	TCTATGTATGCCCCAACCTTCATGATGGTGGTG
A129W	TCTATGTATGCCCCATGGTTCATGATGGTGGTG
A129Y	TCTATGTATGCCCCATACTTCATGATGGTGGTG
A129V	TCTATGTATGCCCCAGTGTTCATGATGGTGGTG
F130A	ATGTATGCCCCAGCTGCCATGATGGTGGTGATT
F130R	ATGTATGCCCCAGCTAGAATGATGGTGGTGATT
F130N	ATGTATGCCCCAGCTAACATGATGGTGGTGATT
F130D	ATGTATGCCCCAGCTGACATGATGGTGGTGATT
F130C	ATGTATGCCCCAGCTTGCATGATGGTGGTGATT
F130Q	ATGTATGCCCCAGCTCAGATGATGGTGGTGATT
F130E	ATGTATGCCCCAGCTGAGATGATGGTGGTGATT
F130G	ATGTATGCCCCAGCTGGCATGATGGTGGTGATT
F130H	ATGTATGCCCCAGCTCACATGATGGTGGTGATT
F130I	ATGTATGCCCCAGCTATCATGATGGTGGTGATT
F130L	ATGTATGCCCCAGCTCTGATGATGGTGGTGATT
F130K	ATGTATGCCCCAGCTAAGATGATGGTGGTGATT
F130M	ATGTATGCCCCAGCTATGATGATGGTGGTGATT
F130P	ATGTATGCCCCAGCTCCCATGATGGTGGTGATT
F130S	ATGTATGCCCCAGCTAGCATGATGGTGGTGATT
F130T	ATGTATGCCCCAGCTACCATGATGGTGGTGATT
F130W	ATGTATGCCCCAGCTTGGATGATGGTGGTGATT
F130Y	ATGTATGCCCCAGCTTACATGATGGTGGTGATT
F130V	ATGTATGCCCCAGCTGTGATGATGGTGGTGATT
M132A	GCCCCAGCTTTCATGGCCGTGGTGATTAGCCTG
M132R	GCCCCAGCTTTCATGAGAGTGGTGATTAGCCTG
M132N	GCCCCAGCTTTCATGAACGTGGTGATTAGCCTG
M132D	GCCCCAGCTTTCATGGACGTGGTGATTAGCCTG
M132C	GCCCCAGCTTTCATGTGCGTGGTGATTAGCCTG
M132Q	GCCCCAGCTTTCATGCAGGTGGTGATTAGCCTG
M132E	GCCCCAGCTTTCATGGAGGTGGTGATTAGCCTG
M132G	GCCCCAGCTTTCATGGGCGTGGTGATTAGCCTG

M132H	GCCCCAGCTTTCATGCACGTGGTGATTAGCCTG
M132I	GCCCCAGCTTTCATGATCGTGGTGATTAGCCTG
M132L	GCCCCAGCTTTCATGCTGGTGGTGATTAGCCTG
M132K	GCCCCAGCTTTCATGAAGGTGGTGATTAGCCTG
M132F	GCCCCAGCTTTCATGTTCGTGGTGATTAGCCTG
M132P	GCCCCAGCTTTCATGCCCGTGGTGATTAGCCTG
M132S	GCCCCAGCTTTCATGAGCGTGGTGATTAGCCTG
M132T	GCCCCAGCTTTCATGACCGTGGTGATTAGCCTG
M132W	GCCCCAGCTTTCATGTGGGTGGTGATTAGCCTG
M132Y	GCCCCAGCTTTCATGTACGTGGTGATTAGCCTG
M132V	GCCCCAGCTTTCATGGTGGTGGTGATTAGCCTG
S136A	ATGATGGTGGTGATTGCCCTGGACCGCTCCCTG
S136R	ATGATGGTGGTGATTAGACTGGACCGCTCCCTG
S136N	ATGATGGTGGTGATTAACCTGGACCGCTCCCTG
S136D	ATGATGGTGGTGATTGACCTGGACCGCTCCCTG
S136C	ATGATGGTGGTGATTTGCCTGGACCGCTCCCTG
S136Q	ATGATGGTGGTGATTCAGCTGGACCGCTCCCTG
S136E	ATGATGGTGGTGATTGAGCTGGACCGCTCCCTG
S136G	ATGATGGTGGTGATTGGCCTGGACCGCTCCCTG
S136H	ATGATGGTGGTGATTCACCTGGACCGCTCCCTG
S136I	ATGATGGTGGTGATTATCCTGGACCGCTCCCTG
S136L	ATGATGGTGGTGATTCTGCTGGACCGCTCCCTG
S136K	ATGATGGTGGTGATTAAGCTGGACCGCTCCCTG
S136M	ATGATGGTGGTGATTATGCTGGACCGCTCCCTG
S136F	ATGATGGTGGTGATTTTCCTGGACCGCTCCCTG
S136P	ATGATGGTGGTGATTCCCCTGGACCGCTCCCTG
S136T	ATGATGGTGGTGATTACCCTGGACCGCTCCCTG
S136W	ATGATGGTGGTGATTTGGCTGGACCGCTCCCTG
S136Y	ATGATGGTGGTGATTTACCTGGACCGCTCCCTG
S136V	ATGATGGTGGTGATTGTGCTGGACCGCTCCCTG
L137A	ATGGTGGTGATTAGCGCCGACCGCTCCCTGGCC
L137R	ATGGTGGTGATTAGCAGAGACCGCTCCCTGGCC
L137N	ATGGTGGTGATTAGCAACGACCGCTCCCTGGCC
L137D	ATGGTGGTGATTAGCGACGACCGCTCCCTGGCC
L137C	ATGGTGGTGATTAGCTGCGACCGCTCCCTGGCC
L137Q	ATGGTGGTGATTAGCCAGGACCGCTCCCTGGCC
L137E	ATGGTGGTGATTAGCGAGGACCGCTCCCTGGCC
L137G	ATGGTGGTGATTAGCGGCGACCGCTCCCTGGCC
L137H	ATGGTGGTGATTAGCCACGACCGCTCCCTGGCC
L137I	ATGGTGGTGATTAGCATCGACCGCTCCCTGGCC
L137K	ATGGTGGTGATTAGCAAGGACCGCTCCCTGGCC

L137M	ATGGTGGTGATTAGCATGGACCGCTCCCTGGCC
L137F	ATGGTGGTGATTAGCTTCGACCGCTCCCTGGCC
L137P	ATGGTGGTGATTAGCCCCGACCGCTCCCTGGCC
L137S	ATGGTGGTGATTAGCAGCGACCGCTCCCTGGCC
L137T	ATGGTGGTGATTAGCACCGACCGCTCCCTGGCC
L137W	ATGGTGGTGATTAGCTGGGACCGCTCCCTGGCC
L137Y	ATGGTGGTGATTAGCTACGACCGCTCCCTGGCC
L137V	ATGGTGGTGATTAGCGTGGACCGCTCCCTGGCC
L141A	AGCCTGGACCGCTCCGCCGCCATCACTCAGCCC
L141R	AGCCTGGACCGCTCCAGAGCCATCACTCAGCCC
L141N	AGCCTGGACCGCTCCAACGCCATCACTCAGCCC
L141D	AGCCTGGACCGCTCCGACGCCATCACTCAGCCC
L141C	AGCCTGGACCGCTCCTGCGCCATCACTCAGCCC
L141Q	AGCCTGGACCGCTCCCAGGCCATCACTCAGCCC
L141E	AGCCTGGACCGCTCCGAGGCCATCACTCAGCCC
L141G	AGCCTGGACCGCTCCGGCGCCATCACTCAGCCC
L141H	AGCCTGGACCGCTCCCACGCCATCACTCAGCCC
L141I	AGCCTGGACCGCTCCATCGCCATCACTCAGCCC
L141K	AGCCTGGACCGCTCCAAGGCCATCACTCAGCCC
L141M	AGCCTGGACCGCTCCATGGCCATCACTCAGCCC
L141F	AGCCTGGACCGCTCCTTCGCCATCACTCAGCCC
L141P	AGCCTGGACCGCTCCCCCGCCATCACTCAGCCC
L141S	AGCCTGGACCGCTCCAGCGCCATCACTCAGCCC
L141T	AGCCTGGACCGCTCCACCGCCATCACTCAGCCC
L141W	AGCCTGGACCGCTCCTGGGCCATCACTCAGCCC
L141Y	AGCCTGGACCGCTCCTACGCCATCACTCAGCCC
L141V	AGCCTGGACCGCTCCGTGGCCATCACTCAGCCC
A148R	ATCACTCAGCCCCTTAGAGTACAAAGCAACAGC
A148N	ATCACTCAGCCCCTTAACGTACAAAGCAACAGC
A148D	ATCACTCAGCCCCTTGACGTACAAAGCAACAGC
A148C	ATCACTCAGCCCCTTTGCGTACAAAGCAACAGC
A148Q	ATCACTCAGCCCCTTCAGGTACAAAGCAACAGC
A148E	ATCACTCAGCCCCTTGAGGTACAAAGCAACAGC
A148G	ATCACTCAGCCCCTTGGCGTACAAAGCAACAGC
A148H	ATCACTCAGCCCCTTCACGTACAAAGCAACAGC
A148I	ATCACTCAGCCCCTTATCGTACAAAGCAACAGC
A148L	ATCACTCAGCCCCTTCTGGTACAAAGCAACAGC
A148K	ATCACTCAGCCCCTTAAGGTACAAAGCAACAGC
A148M	ATCACTCAGCCCCTTATGGTACAAAGCAACAGC
A148F	ATCACTCAGCCCCTTTTCGTACAAAGCAACAGC
A148P	ATCACTCAGCCCCTTCCCGTACAAAGCAACAGC

A148S	ATCACTCAGCCCCTTAGCGTACAAAGCAACAGC
A148T	ATCACTCAGCCCCTTACCGTACAAAGCAACAGC
A148W	ATCACTCAGCCCCTTTGGGTACAAAGCAACAGC
A148Y	ATCACTCAGCCCCTTTACGTACAAAGCAACAGC
A148V	ATCACTCAGCCCCTTGTGGTACAAAGCAACAGC
N152A	CTTGCTGTACAAAGCGCCAGCAAGCTTGAACAG
N152R	CTTGCTGTACAAAGCAGAAGCAAGCTTGAACAG
N152D	CTTGCTGTACAAAGCGACAGCAAGCTTGAACAG
N152C	CTTGCTGTACAAAGCTGCAGCAAGCTTGAACAG
N152Q	CTTGCTGTACAAAGCCAGAGCAAGCTTGAACAG
N152E	CTTGCTGTACAAAGCGAGAGCAAGCTTGAACAG
N152G	CTTGCTGTACAAAGCGGCAGCAAGCTTGAACAG
N152H	CTTGCTGTACAAAGCCACAGCAAGCTTGAACAG
N152I	CTTGCTGTACAAAGCATCAGCAAGCTTGAACAG
N152L	CTTGCTGTACAAAGCCTGAGCAAGCTTGAACAG
N152K	CTTGCTGTACAAAGCAAGAGCAAGCTTGAACAG
N152M	CTTGCTGTACAAAGCATGAGCAAGCTTGAACAG
N152F	CTTGCTGTACAAAGCTTCAGCAAGCTTGAACAG
N152P	CTTGCTGTACAAAGCCCCAGCAAGCTTGAACAG
N152S	CTTGCTGTACAAAGCAGCAGCAAGCTTGAACAG
N152T	CTTGCTGTACAAAGCACCAGCAAGCTTGAACAG
N152W	CTTGCTGTACAAAGCTGGAGCAAGCTTGAACAG
N152Y	CTTGCTGTACAAAGCTACAGCAAGCTTGAACAG
N152V	CTTGCTGTACAAAGCGTGAGCAAGCTTGAACAG
E156A	AGCAACAGCAAGCTTGCCCAGTCTATGATCAGC
E156R	AGCAACAGCAAGCTTAGACAGTCTATGATCAGC
E156N	AGCAACAGCAAGCTTAACCAGTCTATGATCAGC
E156D	AGCAACAGCAAGCTTGACCAGTCTATGATCAGC
E156C	AGCAACAGCAAGCTTTGCCAGTCTATGATCAGC
E156Q	AGCAACAGCAAGCTTCAGCAGTCTATGATCAGC
E156G	AGCAACAGCAAGCTTGGCCAGTCTATGATCAGC
E156H	AGCAACAGCAAGCTTCACCAGTCTATGATCAGC
E156I	AGCAACAGCAAGCTTATCCAGTCTATGATCAGC
E156L	AGCAACAGCAAGCTTCTGCAGTCTATGATCAGC
E156K	AGCAACAGCAAGCTTAAGCAGTCTATGATCAGC
E156M	AGCAACAGCAAGCTTATGCAGTCTATGATCAGC
E156F	AGCAACAGCAAGCTTTTCCAGTCTATGATCAGC
E156P	AGCAACAGCAAGCTTCCCCAGTCTATGATCAGC
E156S	AGCAACAGCAAGCTTAGCCAGTCTATGATCAGC
E156T	AGCAACAGCAAGCTTACCCAGTCTATGATCAGC
E156W	AGCAACAGCAAGCTTTGGCAGTCTATGATCAGC

E156Y	AGCAACAGCAAGCTTTACCAGTCTATGATCAGC
E156V	AGCAACAGCAAGCTTGTGCAGTCTATGATCAGC
I160A	CTTGAACAGTCTATGGCCAGCCTGGCCTGGATT
I160R	CTTGAACAGTCTATGAGAAGCCTGGCCTGGATT
I160N	CTTGAACAGTCTATGAACAGCCTGGCCTGGATT
I160D	CTTGAACAGTCTATGGACAGCCTGGCCTGGATT
I160C	CTTGAACAGTCTATGTGCAGCCTGGCCTGGATT
I160Q	CTTGAACAGTCTATGCAGAGCCTGGCCTGGATT
I160E	CTTGAACAGTCTATGGAGAGCCTGGCCTGGATT
I160G	CTTGAACAGTCTATGGGCAGCCTGGCCTGGATT
I160H	CTTGAACAGTCTATGCACAGCCTGGCCTGGATT
I160L	CTTGAACAGTCTATGCTGAGCCTGGCCTGGATT
I160K	CTTGAACAGTCTATGAAGAGCCTGGCCTGGATT
I160M	CTTGAACAGTCTATGATGAGCCTGGCCTGGATT
I160F	CTTGAACAGTCTATGTTCAGCCTGGCCTGGATT
I160P	CTTGAACAGTCTATGCCCAGCCTGGCCTGGATT
I160S	CTTGAACAGTCTATGAGCAGCCTGGCCTGGATT
I160T	CTTGAACAGTCTATGACCAGCCTGGCCTGGATT
I160W	CTTGAACAGTCTATGTGGAGCCTGGCCTGGATT
I160Y	CTTGAACAGTCTATGTACAGCCTGGCCTGGATT
I160V	CTTGAACAGTCTATGGTGAGCCTGGCCTGGATT
A163R	TCTATGATCAGCCTGAGATGGATTCTCAGCATT
A163N	TCTATGATCAGCCTGAACTGGATTCTCAGCATT
A163D	TCTATGATCAGCCTGGACTGGATTCTCAGCATT
A163C	TCTATGATCAGCCTGTGCTGGATTCTCAGCATT
A163Q	TCTATGATCAGCCTGCAGTGGATTCTCAGCATT
A163E	TCTATGATCAGCCTGGAGTGGATTCTCAGCATT
A163G	TCTATGATCAGCCTGGGCTGGATTCTCAGCATT
A163H	TCTATGATCAGCCTGCACTGGATTCTCAGCATT
A163I	TCTATGATCAGCCTGATCTGGATTCTCAGCATT
A163L	TCTATGATCAGCCTGCTGTGGATTCTCAGCATT
A163K	TCTATGATCAGCCTGAAGTGGATTCTCAGCATT
A163M	TCTATGATCAGCCTGATGTGGATTCTCAGCATT
A163F	TCTATGATCAGCCTGTTCTGGATTCTCAGCATT
A163P	TCTATGATCAGCCTGCCCTGGATTCTCAGCATT
A163S	TCTATGATCAGCCTGAGCTGGATTCTCAGCATT
A163T	TCTATGATCAGCCTGACCTGGATTCTCAGCATT
A163W	TCTATGATCAGCCTGTGGTGGATTCTCAGCATT
A163Y	TCTATGATCAGCCTGTACTGGATTCTCAGCATT
A163V	TCTATGATCAGCCTGGTGTGGATTCTCAGCATT
I165A	ATCAGCCTGGCCTGGGCCCTCAGCATTGTCTTT

I165R	ATCAGCCTGGCCTGGAGACTCAGCATTGTCTTT
I165N	ATCAGCCTGGCCTGGAACCTCAGCATTGTCTTT
I165D	ATCAGCCTGGCCTGGGACCTCAGCATTGTCTTT
I165C	ATCAGCCTGGCCTGGTGCCTCAGCATTGTCTTT
I165Q	ATCAGCCTGGCCTGGCAGCTCAGCATTGTCTTT
I165E	ATCAGCCTGGCCTGGGAGCTCAGCATTGTCTTT
I165G	ATCAGCCTGGCCTGGGGCCTCAGCATTGTCTTT
I165H	ATCAGCCTGGCCTGGCACCTCAGCATTGTCTTT
I165L	ATCAGCCTGGCCTGGCTGCTCAGCATTGTCTTT
I165K	ATCAGCCTGGCCTGGAAGCTCAGCATTGTCTTT
I165M	ATCAGCCTGGCCTGGATGCTCAGCATTGTCTTT
I165F	ATCAGCCTGGCCTGGTTCCTCAGCATTGTCTTT
I165P	ATCAGCCTGGCCTGGCCCCTCAGCATTGTCTTT
I165S	ATCAGCCTGGCCTGGAGCCTCAGCATTGTCTTT
I165T	ATCAGCCTGGCCTGGACCCTCAGCATTGTCTTT
I165W	ATCAGCCTGGCCTGGTGGCTCAGCATTGTCTTT
I165Y	ATCAGCCTGGCCTGGTACCTCAGCATTGTCTTT
I165V	ATCAGCCTGGCCTGGGTGCTCAGCATTGTCTTT
I168A	GCCTGGATTCTCAGCGCCGTCTTTGCAGGACCA
I168R	GCCTGGATTCTCAGCAGAGTCTTTGCAGGACCA
I168N	GCCTGGATTCTCAGCAACGTCTTTGCAGGACCA
I168D	GCCTGGATTCTCAGCGACGTCTTTGCAGGACCA
I168C	GCCTGGATTCTCAGCTGCGTCTTTGCAGGACCA
I168Q	GCCTGGATTCTCAGCCAGGTCTTTGCAGGACCA
I168E	GCCTGGATTCTCAGCGAGGTCTTTGCAGGACCA
I168G	GCCTGGATTCTCAGCGGCGTCTTTGCAGGACCA
I168H	GCCTGGATTCTCAGCCACGTCTTTGCAGGACCA
I168L	GCCTGGATTCTCAGCCTGGTCTTTGCAGGACCA
I168K	GCCTGGATTCTCAGCAAGGTCTTTGCAGGACCA
I168M	GCCTGGATTCTCAGCATGGTCTTTGCAGGACCA
I168F	GCCTGGATTCTCAGCTTCGTCTTTGCAGGACCA
I168P	GCCTGGATTCTCAGCCCCGTCTTTGCAGGACCA
I168S	GCCTGGATTCTCAGCAGCGTCTTTGCAGGACCA
I168T	GCCTGGATTCTCAGCACCGTCTTTGCAGGACCA
I168W	GCCTGGATTCTCAGCTGGGTCTTTGCAGGACCA
I168Y	GCCTGGATTCTCAGCTACGTCTTTGCAGGACCA
I168V	GCCTGGATTCTCAGCGTGGTCTTTGCAGGACCA
Q174A	GTCTTTGCAGGACCAGCCTTATATATCTTCAGG
Q174R	GTCTTTGCAGGACCAAGATTATATATCTTCAGG
Q174N	GTCTTTGCAGGACCAAACTTATATATCTTCAGG
Q174D	GTCTTTGCAGGACCAGACTTATATATCTTCAGG

Q174C	GTCTTTGCAGGACCATGCTTATATATCTTCAGG
Q174E	GTCTTTGCAGGACCAGAGTTATATATCTTCAGG
Q174G	GTCTTTGCAGGACCAGGCTTATATATCTTCAGG
Q174H	GTCTTTGCAGGACCACACTTATATATCTTCAGG
Q174I	GTCTTTGCAGGACCAATCTTATATATCTTCAGG
Q174L	GTCTTTGCAGGACCACTGTTATATATCTTCAGG
Q174K	GTCTTTGCAGGACCAAAGTTATATATCTTCAGG
Q174M	GTCTTTGCAGGACCAATGTTATATATCTTCAGG
Q174F	GTCTTTGCAGGACCATTCTTATATATCTTCAGG
Q174P	GTCTTTGCAGGACCACCCTTATATATCTTCAGG
Q174S	GTCTTTGCAGGACCAAGCTTATATATCTTCAGG
Q174T	GTCTTTGCAGGACCAACCTTATATATCTTCAGG
Q174W	GTCTTTGCAGGACCATGGTTATATATCTTCAGG
Q174Y	GTCTTTGCAGGACCATACTTATATATCTTCAGG
Q174V	GTCTTTGCAGGACCAGTGTTATATATCTTCAGG
L175A	TTTGCAGGACCACAGGCCTATATCTTCAGGATG
L175R	TTTGCAGGACCACAGAGATATATCTTCAGGATG
L175N	TTTGCAGGACCACAGAACTATATCTTCAGGATG
L175D	TTTGCAGGACCACAGGACTATATCTTCAGGATG
L175C	TTTGCAGGACCACAGTGCTATATCTTCAGGATG
L175Q	TTTGCAGGACCACAGCAGTATATCTTCAGGATG
L175E	TTTGCAGGACCACAGGAGTATATCTTCAGGATG
L175G	TTTGCAGGACCACAGGGCTATATCTTCAGGATG
L175H	TTTGCAGGACCACAGCACTATATCTTCAGGATG
L175I	TTTGCAGGACCACAGATCTATATCTTCAGGATG
L175K	TTTGCAGGACCACAGAAGTATATCTTCAGGATG
L175M	TTTGCAGGACCACAGATGTATATCTTCAGGATG
L175F	TTTGCAGGACCACAGTTCTATATCTTCAGGATG
L175P	TTTGCAGGACCACAGCCCTATATCTTCAGGATG
L175S	TTTGCAGGACCACAGAGCTATATCTTCAGGATG
L175T	TTTGCAGGACCACAGACCTATATCTTCAGGATG
L175W	TTTGCAGGACCACAGTGGTATATCTTCAGGATG
L175Y	TTTGCAGGACCACAGTACTATATCTTCAGGATG
L175V	TTTGCAGGACCACAGGTGTATATCTTCAGGATG
I177A	GGACCACAGTTATATGCCTTCAGGATGATCTAC
I177R	GGACCACAGTTATATAGATTCAGGATGATCTAC
I177N	GGACCACAGTTATATAACTTCAGGATGATCTAC
I177D	GGACCACAGTTATATGACTTCAGGATGATCTAC
I177C	GGACCACAGTTATATTGCTTCAGGATGATCTAC
I177Q	GGACCACAGTTATATCAGTTCAGGATGATCTAC
I177E	GGACCACAGTTATATGAGTTCAGGATGATCTAC

I177G	GGACCACAGTTATATGGCTTCAGGATGATCTAC
I177H	GGACCACAGTTATATCACTTCAGGATGATCTAC
I177L	GGACCACAGTTATATCTGTTCAGGATGATCTAC
I177K	GGACCACAGTTATATAAGTTCAGGATGATCTAC
I177M	GGACCACAGTTATATATGTTCAGGATGATCTAC
I177F	GGACCACAGTTATATTTCTTCAGGATGATCTAC
I177P	GGACCACAGTTATATCCCTTCAGGATGATCTAC
I177S	GGACCACAGTTATATAGCTTCAGGATGATCTAC
I177T	GGACCACAGTTATATACCTTCAGGATGATCTAC
I177W	GGACCACAGTTATATTGGTTCAGGATGATCTAC
I177Y	GGACCACAGTTATATTACTTCAGGATGATCTAC
I177V	GGACCACAGTTATATGTGTTCAGGATGATCTAC
G188A	CTAGCAGACGGCTCTGCCCCCACAGTCTTCTCG
G188R	CTAGCAGACGGCTCTAGACCCACAGTCTTCTCG
G188N	CTAGCAGACGGCTCTAACCCCACAGTCTTCTCG
G188D	CTAGCAGACGGCTCTGACCCCACAGTCTTCTCG
G188C	CTAGCAGACGGCTCTTGCCCCACAGTCTTCTCG
G188Q	CTAGCAGACGGCTCTCAGCCCACAGTCTTCTCG
G188E	CTAGCAGACGGCTCTGAGCCCACAGTCTTCTCG
G188H	CTAGCAGACGGCTCTCACCCCACAGTCTTCTCG
G188I	CTAGCAGACGGCTCTATCCCCACAGTCTTCTCG
G188L	CTAGCAGACGGCTCTCTGCCCACAGTCTTCTCG
G188K	CTAGCAGACGGCTCTAAGCCCACAGTCTTCTCG
G188M	CTAGCAGACGGCTCTATGCCCACAGTCTTCTCG
G188F	CTAGCAGACGGCTCTTTCCCCACAGTCTTCTCG
G188P	CTAGCAGACGGCTCTCCCCCCACAGTCTTCTCG
G188S	CTAGCAGACGGCTCTAGCCCCACAGTCTTCTCG
G188T	CTAGCAGACGGCTCTACCCCCACAGTCTTCTCG
G188W	CTAGCAGACGGCTCTTGGCCCACAGTCTTCTCG
G188Y	CTAGCAGACGGCTCTTACCCCACAGTCTTCTCG
G188V	CTAGCAGACGGCTCTGTGCCCACAGTCTTCTCG
F192A	TCTGGGCCCACAGTCGCCTCGCAATGTGTGACC
F192R	TCTGGGCCCACAGTCAGATCGCAATGTGTGACC
F192N	TCTGGGCCCACAGTCAACTCGCAATGTGTGACC
F192D	TCTGGGCCCACAGTCGACTCGCAATGTGTGACC
F192C	TCTGGGCCCACAGTCTGCTCGCAATGTGTGACC
F192Q	TCTGGGCCCACAGTCCAGTCGCAATGTGTGACC
F192E	TCTGGGCCCACAGTCGAGTCGCAATGTGTGACC
F192G	TCTGGGCCCACAGTCGGCTCGCAATGTGTGACC
F192H	TCTGGGCCCACAGTCCACTCGCAATGTGTGACC
F192I	TCTGGGCCCACAGTCATCTCGCAATGTGTGACC

F192L	TCTGGGCCCACAGTCCTGTCGCAATGTGTGACC
F192K	TCTGGGCCCACAGTCAAGTCGCAATGTGTGACC
F192M	TCTGGGCCCACAGTCATGTCGCAATGTGTGACC
F192P	TCTGGGCCCACAGTCCCCTCGCAATGTGTGACC
F192S	TCTGGGCCCACAGTCAGCTCGCAATGTGTGACC
F192T	TCTGGGCCCACAGTCACCTCGCAATGTGTGACC
F192W	TCTGGGCCCACAGTCTGGTCGCAATGTGTGACC
F192Y	TCTGGGCCCACAGTCTACTCGCAATGTGTGACC
F192V	TCTGGGCCCACAGTCGTGTCGCAATGTGTGACC
V196A	GTCTTCTCGCAATGTGCCACCCACTGCAGCTTT
V196R	GTCTTCTCGCAATGTAGAACCCACTGCAGCTTT
V196N	GTCTTCTCGCAATGTAACACCCACTGCAGCTTT
V196D	GTCTTCTCGCAATGTGACACCCACTGCAGCTTT
V196C	GTCTTCTCGCAATGTTGCACCCACTGCAGCTTT
V196Q	GTCTTCTCGCAATGTCAGACCCACTGCAGCTTT
V196E	GTCTTCTCGCAATGTGAGACCCACTGCAGCTTT
V196G	GTCTTCTCGCAATGTGGCACCCACTGCAGCTTT
V196H	GTCTTCTCGCAATGTCACACCCACTGCAGCTTT
V196I	GTCTTCTCGCAATGTATCACCCACTGCAGCTTT
V196L	GTCTTCTCGCAATGTCTGACCCACTGCAGCTTT
V196K	GTCTTCTCGCAATGTAAGACCCACTGCAGCTTT
V196M	GTCTTCTCGCAATGTATGACCCACTGCAGCTTT
V196F	GTCTTCTCGCAATGTTTCACCCACTGCAGCTTT
V196P	GTCTTCTCGCAATGTCCCACCCACTGCAGCTTT
V196S	GTCTTCTCGCAATGTAGCACCCACTGCAGCTTT
V196T	GTCTTCTCGCAATGTACCACCCACTGCAGCTTT
V196W	GTCTTCTCGCAATGTTGGACCCACTGCAGCTTT
V196Y	GTCTTCTCGCAATGTTACACCCACTGCAGCTTT
S200A	TGTGTGACCCACTGCGCCTTTCCACAGTGGTGG
S200R	TGTGTGACCCACTGCAGATTTCCACAGTGGTGG
S200N	TGTGTGACCCACTGCAACTTTCCACAGTGGTGG
S200D	TGTGTGACCCACTGCGACTTTCCACAGTGGTGG
S200C	TGTGTGACCCACTGCTGCTTTCCACAGTGGTGG
S200Q	TGTGTGACCCACTGCCAGTTTCCACAGTGGTGG
S200E	TGTGTGACCCACTGCGAGTTTCCACAGTGGTGG
S200G	TGTGTGACCCACTGCGGCTTTCCACAGTGGTGG
S200H	TGTGTGACCCACTGCCACTTTCCACAGTGGTGG
S200I	TGTGTGACCCACTGCATCTTTCCACAGTGGTGG
S200L	TGTGTGACCCACTGCCTGTTTCCACAGTGGTGG
S200K	TGTGTGACCCACTGCAAGTTTCCACAGTGGTGG
S200M	TGTGTGACCCACTGCATGTTTCCACAGTGGTGG

S200F	TGTGTGACCCACTGCTTCTTTCCACAGTGGTGG
S200P	TGTGTGACCCACTGCCCCTTTCCACAGTGGTGG
S200T	TGTGTGACCCACTGCACCTTTCCACAGTGGTGG
S200W	TGTGTGACCCACTGCTGGTTTCCACAGTGGTGG
S200Y	TGTGTGACCCACTGCTACTTTCCACAGTGGTGG
S200V	TGTGTGACCCACTGCGTGTTTCCACAGTGGTGG
W204A	TGCAGCTTTCCACAGGCCTGGCATCAGGCCTTC
W204R	TGCAGCTTTCCACAGAGATGGCATCAGGCCTTC
W204N	TGCAGCTTTCCACAGAACTGGCATCAGGCCTTC
W204D	TGCAGCTTTCCACAGGACTGGCATCAGGCCTTC
W204C	TGCAGCTTTCCACAGTGCTGGCATCAGGCCTTC
W204Q	TGCAGCTTTCCACAGCAGTGGCATCAGGCCTTC
W204E	TGCAGCTTTCCACAGGAGTGGCATCAGGCCTTC
W204G	TGCAGCTTTCCACAGGGCTGGCATCAGGCCTTC
W204H	TGCAGCTTTCCACAGCACTGGCATCAGGCCTTC
W204I	TGCAGCTTTCCACAGATCTGGCATCAGGCCTTC
W204L	TGCAGCTTTCCACAGCTGTGGCATCAGGCCTTC
W204K	TGCAGCTTTCCACAGAAGTGGCATCAGGCCTTC
W204M	TGCAGCTTTCCACAGATGTGGCATCAGGCCTTC
W204F	TGCAGCTTTCCACAGTTCTGGCATCAGGCCTTC
W204P	TGCAGCTTTCCACAGCCCTGGCATCAGGCCTTC
W204S	TGCAGCTTTCCACAGAGCTGGCATCAGGCCTTC
W204T	TGCAGCTTTCCACAGACCTGGCATCAGGCCTTC
W204Y	TGCAGCTTTCCACAGTACTGGCATCAGGCCTTC
W204V	TGCAGCTTTCCACAGGTGTGGCATCAGGCCTTC
A208R	CAGTGGTGGCATCAGAGATTCTACAACTTTTTC
A208N	CAGTGGTGGCATCAGAACTTCTACAACTTTTTC
A208D	CAGTGGTGGCATCAGGACTTCTACAACTTTTTC
A208C	CAGTGGTGGCATCAGTGCTTCTACAACTTTTTC
A208Q	CAGTGGTGGCATCAGCAGTTCTACAACTTTTTC
A208E	CAGTGGTGGCATCAGGAGTTCTACAACTTTTTC
A208G	CAGTGGTGGCATCAGGGCTTCTACAACTTTTTC
A208H	CAGTGGTGGCATCAGCACTTCTACAACTTTTTC
A208I	CAGTGGTGGCATCAGATCTTCTACAACTTTTTC
A208L	CAGTGGTGGCATCAGCTGTTCTACAACTTTTTC
A208K	CAGTGGTGGCATCAGAAGTTCTACAACTTTTTC
A208M	CAGTGGTGGCATCAGATGTTCTACAACTTTTTC
A208F	CAGTGGTGGCATCAGTTCTTCTACAACTTTTTC
A208P	CAGTGGTGGCATCAGCCCTTCTACAACTTTTTC
A208S	CAGTGGTGGCATCAGAGCTTCTACAACTTTTTC
A208T	CAGTGGTGGCATCAGACCTTCTACAACTTTTTC

A208W	CAGTGGTGGCATCAGTGGTTCTACAACTTTTTC
A208Y	CAGTGGTGGCATCAGTACTTCTACAACTTTTTC
A208V	CAGTGGTGGCATCAGGTGTTCTACAACTTTTTC
F219A	ACCTTCGGCTGCCTCGCCATCATCCCCCTCCTC
F219R	ACCTTCGGCTGCCTCAGAATCATCCCCCTCCTC
F219N	ACCTTCGGCTGCCTCAACATCATCCCCCTCCTC
F219D	ACCTTCGGCTGCCTCGACATCATCCCCCTCCTC
F219C	ACCTTCGGCTGCCTCTGCATCATCCCCCTCCTC
F219Q	ACCTTCGGCTGCCTCCAGATCATCCCCCTCCTC
F219E	ACCTTCGGCTGCCTCGAGATCATCCCCCTCCTC
F219G	ACCTTCGGCTGCCTCGGCATCATCCCCCTCCTC
F219H	ACCTTCGGCTGCCTCCACATCATCCCCCTCCTC
F219I	ACCTTCGGCTGCCTCATCATCATCCCCCTCCTC
F219L	ACCTTCGGCTGCCTCCTGATCATCCCCCTCCTC
F219K	ACCTTCGGCTGCCTCAAGATCATCCCCCTCCTC
F219M	ACCTTCGGCTGCCTCATGATCATCCCCCTCCTC
F219P	ACCTTCGGCTGCCTCCCCATCATCCCCCTCCTC
F219S	ACCTTCGGCTGCCTCAGCATCATCCCCCTCCTC
F219T	ACCTTCGGCTGCCTCACCATCATCCCCCTCCTC
F219W	ACCTTCGGCTGCCTCTGGATCATCCCCCTCCTC
F219Y	ACCTTCGGCTGCCTCTACATCATCCCCCTCCTC
F219V	ACCTTCGGCTGCCTCGTGATCATCCCCCTCCTC
I221A	GGCTGCCTCTTCATCGCCCCCCTCCTCATCATG
I221R	GGCTGCCTCTTCATCAGACCCCTCCTCATCATG
I221N	GGCTGCCTCTTCATCAACCCCCTCCTCATCATG
I221D	GGCTGCCTCTTCATCGACCCCCTCCTCATCATG
I221C	GGCTGCCTCTTCATCTGCCCCCTCCTCATCATG
I221Q	GGCTGCCTCTTCATCCAGCCCCTCCTCATCATG
I221E	GGCTGCCTCTTCATCGAGCCCCTCCTCATCATG
I221G	GGCTGCCTCTTCATCGGCCCCCTCCTCATCATG
I221H	GGCTGCCTCTTCATCCACCCCCTCCTCATCATG
I221L	GGCTGCCTCTTCATCCTGCCCCTCCTCATCATG
I221K	GGCTGCCTCTTCATCAAGCCCCTCCTCATCATG
I221M	GGCTGCCTCTTCATCATGCCCCTCCTCATCATG
I221F	GGCTGCCTCTTCATCTTCCCCCTCCTCATCATG
I221P	GGCTGCCTCTTCATCCCCCCCCTCCTCATCATG
I221S	GGCTGCCTCTTCATCAGCCCCCTCCTCATCATG
I221T	GGCTGCCTCTTCATCACCCCCCTCCTCATCATG
I221W	GGCTGCCTCTTCATCTGGCCCCTCCTCATCATG
I221Y	GGCTGCCTCTTCATCTACCCCCTCCTCATCATG
I221V	GGCTGCCTCTTCATCGTGCCCCTCCTCATCATG

M226A	ATCCCCCTCCTCATCGCCCTAATCTGCAATGCC
M226R	ATCCCCCTCCTCATCAGACTAATCTGCAATGCC
M226N	ATCCCCCTCCTCATCAACCTAATCTGCAATGCC
M226D	ATCCCCCTCCTCATCGACCTAATCTGCAATGCC
M226C	ATCCCCCTCCTCATCTGCCTAATCTGCAATGCC
M226Q	ATCCCCCTCCTCATCCAGCTAATCTGCAATGCC
M226E	ATCCCCCTCCTCATCGAGCTAATCTGCAATGCC
M226G	ATCCCCCTCCTCATCGGCCTAATCTGCAATGCC
M226H	ATCCCCCTCCTCATCCACCTAATCTGCAATGCC
M226I	ATCCCCCTCCTCATCATCCTAATCTGCAATGCC
M226L	ATCCCCCTCCTCATCCTGCTAATCTGCAATGCC
M226K	ATCCCCCTCCTCATCAAGCTAATCTGCAATGCC
M226F	ATCCCCCTCCTCATCTTCCTAATCTGCAATGCC
M226P	ATCCCCCTCCTCATCCCCCTAATCTGCAATGCC
M226S	ATCCCCCTCCTCATCAGCCTAATCTGCAATGCC
M226T	ATCCCCCTCCTCATCACCCTAATCTGCAATGCC
M226W	ATCCCCCTCCTCATCTGGCTAATCTGCAATGCC
M226Y	ATCCCCCTCCTCATCTACCTAATCTGCAATGCC
M226V	ATCCCCCTCCTCATCGTGCTAATCTGCAATGCC
I228A	CTCCTCATCATGCTAGCCTGCAATGCCAAAATC
I228R	CTCCTCATCATGCTAAGATGCAATGCCAAAATC
I228N	CTCCTCATCATGCTAAACTGCAATGCCAAAATC
I228D	CTCCTCATCATGCTAGACTGCAATGCCAAAATC
I228C	CTCCTCATCATGCTATGCTGCAATGCCAAAATC
I228Q	CTCCTCATCATGCTACAGTGCAATGCCAAAATC
I228E	CTCCTCATCATGCTAGAGTGCAATGCCAAAATC
I228G	CTCCTCATCATGCTAGGCTGCAATGCCAAAATC
I228H	CTCCTCATCATGCTACACTGCAATGCCAAAATC
I228L	CTCCTCATCATGCTACTGTGCAATGCCAAAATC
I228K	CTCCTCATCATGCTAAAGTGCAATGCCAAAATC
I228M	CTCCTCATCATGCTAATGTGCAATGCCAAAATC
I228F	CTCCTCATCATGCTATTCTGCAATGCCAAAATC
I228P	CTCCTCATCATGCTACCCTGCAATGCCAAAATC
I228S	CTCCTCATCATGCTAAGCTGCAATGCCAAAATC
I228T	CTCCTCATCATGCTAACCTGCAATGCCAAAATC
I228W	CTCCTCATCATGCTATGGTGCAATGCCAAAATC
I228Y	CTCCTCATCATGCTATACTGCAATGCCAAAATC
I228V	CTCCTCATCATGCTAGTGTGCAATGCCAAAATC
N230A	ATCATGCTAATCTGCGCCGCCAAAATCATCTTT
N230R	ATCATGCTAATCTGCAGAGCCAAAATCATCTTT
N230D	ATCATGCTAATCTGCGACGCCAAAATCATCTTT

N230C	ATCATGCTAATCTGCTGCGCCAAAATCATCTTT
N230Q	ATCATGCTAATCTGCCAGGCCAAAATCATCTTT
N230E	ATCATGCTAATCTGCGAGGCCAAAATCATCTTT
N230G	ATCATGCTAATCTGCGGCGCCAAAATCATCTTT
N230H	ATCATGCTAATCTGCCACGCCAAAATCATCTTT
N230I	ATCATGCTAATCTGCATCGCCAAAATCATCTTT
N230L	ATCATGCTAATCTGCCTGGCCAAAATCATCTTT
N230K	ATCATGCTAATCTGCAAGGCCAAAATCATCTTT
N230M	ATCATGCTAATCTGCATGGCCAAAATCATCTTT
N230F	ATCATGCTAATCTGCTTCGCCAAAATCATCTTT
N230P	ATCATGCTAATCTGCCCCGCCAAAATCATCTTT
N230S	ATCATGCTAATCTGCAGCGCCAAAATCATCTTT
N230T	ATCATGCTAATCTGCACCGCCAAAATCATCTTT
N230W	ATCATGCTAATCTGCTGGGCCAAAATCATCTTT
N230Y	ATCATGCTAATCTGCTACGCCAAAATCATCTTT
N230V	ATCATGCTAATCTGCGTGGCCAAAATCATCTTT
K232A	CTAATCTGCAATGCCGCCATCATCTTTGCTCTC
K232R	CTAATCTGCAATGCCAGAATCATCTTTGCTCTC
K232N	CTAATCTGCAATGCCAACATCATCTTTGCTCTC
K232D	CTAATCTGCAATGCCGACATCATCTTTGCTCTC
K232C	CTAATCTGCAATGCCTGCATCATCTTTGCTCTC
K232Q	CTAATCTGCAATGCCCAGATCATCTTTGCTCTC
K232E	CTAATCTGCAATGCCGAGATCATCTTTGCTCTC
K232G	CTAATCTGCAATGCCGGCATCATCTTTGCTCTC
K232H	CTAATCTGCAATGCCCACATCATCTTTGCTCTC
K232I	CTAATCTGCAATGCCATCATCATCTTTGCTCTC
K232L	CTAATCTGCAATGCCCTGATCATCTTTGCTCTC
K232M	CTAATCTGCAATGCCATGATCATCTTTGCTCTC
K232F	CTAATCTGCAATGCCTTCATCATCTTTGCTCTC
K232P	CTAATCTGCAATGCCCCCATCATCTTTGCTCTC
K232S	CTAATCTGCAATGCCAGCATCATCTTTGCTCTC
K232T	CTAATCTGCAATGCCACCATCATCTTTGCTCTC
K232W	CTAATCTGCAATGCCTGGATCATCTTTGCTCTC
K232Y	CTAATCTGCAATGCCTACATCATCTTTGCTCTC
K232V	CTAATCTGCAATGCCGTGATCATCTTTGCTCTC
T238A	ATCATCTTTGCTCTCGCCCGAGTCCTTCATCAA
T238R	ATCATCTTTGCTCTCAGACGAGTCCTTCATCAA
T238N	ATCATCTTTGCTCTCAACCGAGTCCTTCATCAA
T238D	ATCATCTTTGCTCTCGACCGAGTCCTTCATCAA
T238C	ATCATCTTTGCTCTCTGCCGAGTCCTTCATCAA
T238Q	ATCATCTTTGCTCTCCAGCGAGTCCTTCATCAA

T238E	ATCATCTTTGCTCTCGAGCGAGTCCTTCATCAA
T238G	ATCATCTTTGCTCTCGGCCGAGTCCTTCATCAA
T238H	ATCATCTTTGCTCTCCACCGAGTCCTTCATCAA
T238I	ATCATCTTTGCTCTCATCCGAGTCCTTCATCAA
T238L	ATCATCTTTGCTCTCCTGCGAGTCCTTCATCAA
T238K	ATCATCTTTGCTCTCAAGCGAGTCCTTCATCAA
T238M	ATCATCTTTGCTCTCATGCGAGTCCTTCATCAA
T238F	ATCATCTTTGCTCTCTTCCGAGTCCTTCATCAA
T238P	ATCATCTTTGCTCTCCCCCGAGTCCTTCATCAA
T238S	ATCATCTTTGCTCTCAGCCGAGTCCTTCATCAA
T238W	ATCATCTTTGCTCTCTGGCGAGTCCTTCATCAA
T238Y	ATCATCTTTGCTCTCTACCGAGTCCTTCATCAA
T238V	ATCATCTTTGCTCTCGTGCGAGTCCTTCATCAA
H242A	CTCACGCGAGTCCTTGCCCAAGACCCACGCAAA
H242R	CTCACGCGAGTCCTTAGACAAGACCCACGCAAA
H242N	CTCACGCGAGTCCTTAACCAAGACCCACGCAAA
H242D	CTCACGCGAGTCCTTGACCAAGACCCACGCAAA
H242C	CTCACGCGAGTCCTTTGCCAAGACCCACGCAAA
H242Q	CTCACGCGAGTCCTTCAGCAAGACCCACGCAAA
H242E	CTCACGCGAGTCCTTGAGCAAGACCCACGCAAA
H242G	CTCACGCGAGTCCTTGGCCAAGACCCACGCAAA
H242I	CTCACGCGAGTCCTTATCCAAGACCCACGCAAA
H242L	CTCACGCGAGTCCTTCTGCAAGACCCACGCAAA
H242K	CTCACGCGAGTCCTTAAGCAAGACCCACGCAAA
H242M	CTCACGCGAGTCCTTATGCAAGACCCACGCAAA
H242F	CTCACGCGAGTCCTTTTCCAAGACCCACGCAAA
H242P	CTCACGCGAGTCCTTCCCCAAGACCCACGCAAA
H242S	CTCACGCGAGTCCTTAGCCAAGACCCACGCAAA
H242T	CTCACGCGAGTCCTTACCCAAGACCCACGCAAA
H242W	CTCACGCGAGTCCTTTGGCAAGACCCACGCAAA
H242Y	CTCACGCGAGTCCTTTACCAAGACCCACGCAAA
H242V	CTCACGCGAGTCCTTGTGCAAGACCCACGCAAA
R246A	CTTCATCAAGACCCAGCCAAACTACAGCTGAAT
R246N	CTTCATCAAGACCCAAACAAACTACAGCTGAAT
R246D	CTTCATCAAGACCCAGACAAACTACAGCTGAAT
R246C	CTTCATCAAGACCCATGCAAACTACAGCTGAAT
R246Q	CTTCATCAAGACCCACAGAAACTACAGCTGAAT
R246E	CTTCATCAAGACCCAGAGAAACTACAGCTGAAT
R246G	CTTCATCAAGACCCAGGCAAACTACAGCTGAAT
R246H	CTTCATCAAGACCCACACAAACTACAGCTGAAT
R246I	CTTCATCAAGACCCAATCAAACTACAGCTGAAT

R246L	CTTCATCAAGACCCACTGAAACTACAGCTGAAT
R246K	CTTCATCAAGACCCAAAGAAACTACAGCTGAAT
R246M	CTTCATCAAGACCCAATGAAACTACAGCTGAAT
R246F	CTTCATCAAGACCCATTCAAACTACAGCTGAAT
R246P	CTTCATCAAGACCCACCCAAACTACAGCTGAAT
R246S	CTTCATCAAGACCCAAGCAAACTACAGCTGAAT
R246T	CTTCATCAAGACCCAACCAAACTACAGCTGAAT
R246W	CTTCATCAAGACCCATGGAAACTACAGCTGAAT
R246Y	CTTCATCAAGACCCATACAAACTACAGCTGAAT
R246V	CTTCATCAAGACCCAGTGAAACTACAGCTGAAT
L250A	CCACGCAAACTACAGGCCAATCAGTCCAAGAAT
L250R	CCACGCAAACTACAGAGAAATCAGTCCAAGAAT
L250N	CCACGCAAACTACAGAACAATCAGTCCAAGAAT
L250D	CCACGCAAACTACAGGACAATCAGTCCAAGAAT
L250C	CCACGCAAACTACAGTGCAATCAGTCCAAGAAT
L250Q	CCACGCAAACTACAGCAGAATCAGTCCAAGAAT
L250E	CCACGCAAACTACAGGAGAATCAGTCCAAGAAT
L250G	CCACGCAAACTACAGGGCAATCAGTCCAAGAAT
L250H	CCACGCAAACTACAGCACAATCAGTCCAAGAAT
L250I	CCACGCAAACTACAGATCAATCAGTCCAAGAAT
L250K	CCACGCAAACTACAGAAGAATCAGTCCAAGAAT
L250M	CCACGCAAACTACAGATGAATCAGTCCAAGAAT
L250F	CCACGCAAACTACAGTTCAATCAGTCCAAGAAT
L250P	CCACGCAAACTACAGCCCAATCAGTCCAAGAAT
L250S	CCACGCAAACTACAGAGCAATCAGTCCAAGAAT
L250T	CCACGCAAACTACAGACCAATCAGTCCAAGAAT
L250W	CCACGCAAACTACAGTGGAATCAGTCCAAGAAT
L250Y	CCACGCAAACTACAGTACAATCAGTCCAAGAAT
L250V	CCACGCAAACTACAGGTGAATCAGTCCAAGAAT
K254A	CAGCTGAATCAGTCCGCCAATAATATCCCAAGA
K254R	CAGCTGAATCAGTCCAGAAATAATATCCCAAGA
K254N	CAGCTGAATCAGTCCAACAATAATATCCCAAGA
K254D	CAGCTGAATCAGTCCGACAATAATATCCCAAGA
K254C	CAGCTGAATCAGTCCTGCAATAATATCCCAAGA
K254Q	CAGCTGAATCAGTCCCAGAATAATATCCCAAGA
K254E	CAGCTGAATCAGTCCGAGAATAATATCCCAAGA
K254G	CAGCTGAATCAGTCCGGCAATAATATCCCAAGA
K254H	CAGCTGAATCAGTCCCACAATAATATCCCAAGA
K254I	CAGCTGAATCAGTCCATCAATAATATCCCAAGA
K254L	CAGCTGAATCAGTCCCTGAATAATATCCCAAGA
K254M	CAGCTGAATCAGTCCATGAATAATATCCCAAGA

K254F	CAGCTGAATCAGTCCTTCAATAATATCCCAAGA
K254P	CAGCTGAATCAGTCCCCCAATAATATCCCAAGA
K254S	CAGCTGAATCAGTCCAGCAATAATATCCCAAGA
K254T	CAGCTGAATCAGTCCACCAATAATATCCCAAGA
K254W	CAGCTGAATCAGTCCTGGAATAATATCCCAAGA
K254Y	CAGCTGAATCAGTCCTACAATAATATCCCAAGA
K254V	CAGCTGAATCAGTCCGTGAATAATATCCCAAGA
P258A	TCCAAGAATAATATCGCCAGAGCTCGGCTGAGA
P258R	TCCAAGAATAATATCAGAAGAGCTCGGCTGAGA
P258N	TCCAAGAATAATATCAACAGAGCTCGGCTGAGA
P258D	TCCAAGAATAATATCGACAGAGCTCGGCTGAGA
P258C	TCCAAGAATAATATCTGCAGAGCTCGGCTGAGA
P258Q	TCCAAGAATAATATCCAGAGAGCTCGGCTGAGA
P258E	TCCAAGAATAATATCGAGAGAGCTCGGCTGAGA
P258G	TCCAAGAATAATATCGGCAGAGCTCGGCTGAGA
P258H	TCCAAGAATAATATCCACAGAGCTCGGCTGAGA
P258I	TCCAAGAATAATATCATCAGAGCTCGGCTGAGA
P258L	TCCAAGAATAATATCCTGAGAGCTCGGCTGAGA
P258K	TCCAAGAATAATATCAAGAGAGCTCGGCTGAGA
P258M	TCCAAGAATAATATCATGAGAGCTCGGCTGAGA
P258F	TCCAAGAATAATATCTTCAGAGCTCGGCTGAGA
P258S	TCCAAGAATAATATCAGCAGAGCTCGGCTGAGA
P258T	TCCAAGAATAATATCACCAGAGCTCGGCTGAGA
P258W	TCCAAGAATAATATCTGGAGAGCTCGGCTGAGA
P258Y	TCCAAGAATAATATCTACAGAGCTCGGCTGAGA
P258V	TCCAAGAATAATATCGTGAGAGCTCGGCTGAGA
L262A	ATCCCAAGAGCTCGGGCCAGAACGCTAAAGATG
L262R	ATCCCAAGAGCTCGGAGAAGAACGCTAAAGATG
L262N	ATCCCAAGAGCTCGGAACAGAACGCTAAAGATG
L262D	ATCCCAAGAGCTCGGGACAGAACGCTAAAGATG
L262C	ATCCCAAGAGCTCGGTGCAGAACGCTAAAGATG
L262Q	ATCCCAAGAGCTCGGCAGAGAACGCTAAAGATG
L262E	ATCCCAAGAGCTCGGGAGAGAACGCTAAAGATG
L262G	ATCCCAAGAGCTCGGGGCAGAACGCTAAAGATG
L262H	ATCCCAAGAGCTCGGCACAGAACGCTAAAGATG
L262I	ATCCCAAGAGCTCGGATCAGAACGCTAAAGATG
L262K	ATCCCAAGAGCTCGGAAGAGAACGCTAAAGATG
L262M	ATCCCAAGAGCTCGGATGAGAACGCTAAAGATG
L262F	ATCCCAAGAGCTCGGTTCAGAACGCTAAAGATG
L262P	ATCCCAAGAGCTCGGCCCAGAACGCTAAAGATG
L262S	ATCCCAAGAGCTCGGAGCAGAACGCTAAAGATG

L262T	ATCCCAAGAGCTCGGACCAGAACGCTAAAGATG
L262W	ATCCCAAGAGCTCGGTGGAGAACGCTAAAGATG
L262Y	ATCCCAAGAGCTCGGTACAGAACGCTAAAGATG
L262V	ATCCCAAGAGCTCGGGTGAGAACGCTAAAGATG
K266A	CGGCTGAGAACGCTAGCCATGACAGTCGCATTC
K266R	CGGCTGAGAACGCTAAGAATGACAGTCGCATTC
K266N	CGGCTGAGAACGCTAAACATGACAGTCGCATTC
K266D	CGGCTGAGAACGCTAGACATGACAGTCGCATTC
K266C	CGGCTGAGAACGCTATGCATGACAGTCGCATTC
K266Q	CGGCTGAGAACGCTACAGATGACAGTCGCATTC
K266E	CGGCTGAGAACGCTAGAGATGACAGTCGCATTC
K266G	CGGCTGAGAACGCTAGGCATGACAGTCGCATTC
K266H	CGGCTGAGAACGCTACACATGACAGTCGCATTC
K266I	CGGCTGAGAACGCTAATCATGACAGTCGCATTC
K266L	CGGCTGAGAACGCTACTGATGACAGTCGCATTC
K266M	CGGCTGAGAACGCTAATGATGACAGTCGCATTC
K266F	CGGCTGAGAACGCTATTCATGACAGTCGCATTC
K266P	CGGCTGAGAACGCTACCCATGACAGTCGCATTC
K266S	CGGCTGAGAACGCTAAGCATGACAGTCGCATTC
K266T	CGGCTGAGAACGCTAACCATGACAGTCGCATTC
K266W	CGGCTGAGAACGCTATGGATGACAGTCGCATTC
K266Y	CGGCTGAGAACGCTATACATGACAGTCGCATTC
K266V	CGGCTGAGAACGCTAGTGATGACAGTCGCATTC
F271A	AAGATGACAGTCGCAGCCGCTACCTCCTTTGTC
F271R	AAGATGACAGTCGCAAGAGCTACCTCCTTTGTC
F271N	AAGATGACAGTCGCAAACGCTACCTCCTTTGTC
F271D	AAGATGACAGTCGCAGACGCTACCTCCTTTGTC
F271C	AAGATGACAGTCGCATGCGCTACCTCCTTTGTC
F271Q	AAGATGACAGTCGCACAGGCTACCTCCTTTGTC
F271E	AAGATGACAGTCGCAGAGGCTACCTCCTTTGTC
F271G	AAGATGACAGTCGCAGGCGCTACCTCCTTTGTC
F271H	AAGATGACAGTCGCACACGCTACCTCCTTTGTC
F271I	AAGATGACAGTCGCAATCGCTACCTCCTTTGTC
F271L	AAGATGACAGTCGCACTGGCTACCTCCTTTGTC
F271K	AAGATGACAGTCGCAAAGGCTACCTCCTTTGTC
F271M	AAGATGACAGTCGCAATGGCTACCTCCTTTGTC
F271P	AAGATGACAGTCGCACCCGCTACCTCCTTTGTC
F271S	AAGATGACAGTCGCAAGCGCTACCTCCTTTGTC
F271T	AAGATGACAGTCGCAACCGCTACCTCCTTTGTC
F271W	AAGATGACAGTCGCATGGGCTACCTCCTTTGTC
F271Y	AAGATGACAGTCGCATACGCTACCTCCTTTGTC

F271V	AAGATGACAGTCGCAGTGGCTACCTCCTTTGTC
T273A	ACAGTCGCATTCGCTGCCTCCTTTGTCGTCTGC
T273R	ACAGTCGCATTCGCTAGATCCTTTGTCGTCTGC
T273N	ACAGTCGCATTCGCTAACTCCTTTGTCGTCTGC
T273D	ACAGTCGCATTCGCTGACTCCTTTGTCGTCTGC
T273C	ACAGTCGCATTCGCTTGCTCCTTTGTCGTCTGC
T273Q	ACAGTCGCATTCGCTCAGTCCTTTGTCGTCTGC
T273E	ACAGTCGCATTCGCTGAGTCCTTTGTCGTCTGC
T273G	ACAGTCGCATTCGCTGGCTCCTTTGTCGTCTGC
T273H	ACAGTCGCATTCGCTCACTCCTTTGTCGTCTGC
T273I	ACAGTCGCATTCGCTATCTCCTTTGTCGTCTGC
T273L	ACAGTCGCATTCGCTCTGTCCTTTGTCGTCTGC
T273K	ACAGTCGCATTCGCTAAGTCCTTTGTCGTCTGC
T273M	ACAGTCGCATTCGCTATGTCCTTTGTCGTCTGC
T273F	ACAGTCGCATTCGCTTTCTCCTTTGTCGTCTGC
T273P	ACAGTCGCATTCGCTCCCTCCTTTGTCGTCTGC
T273S	ACAGTCGCATTCGCTAGCTCCTTTGTCGTCTGC
T273W	ACAGTCGCATTCGCTTGGTCCTTTGTCGTCTGC
T273Y	ACAGTCGCATTCGCTTACTCCTTTGTCGTCTGC
T273V	ACAGTCGCATTCGCTGTGTCCTTTGTCGTCTGC
F275A	GCATTCGCTACCTCCGCCGTCGTCTGCTGGACT
F275R	GCATTCGCTACCTCCAGAGTCGTCTGCTGGACT
F275N	GCATTCGCTACCTCCAACGTCGTCTGCTGGACT
F275D	GCATTCGCTACCTCCGACGTCGTCTGCTGGACT
F275C	GCATTCGCTACCTCCTGCGTCGTCTGCTGGACT
F275Q	GCATTCGCTACCTCCCAGGTCGTCTGCTGGACT
F275E	GCATTCGCTACCTCCGAGGTCGTCTGCTGGACT
F275G	GCATTCGCTACCTCCGGCGTCGTCTGCTGGACT
F275H	GCATTCGCTACCTCCCACGTCGTCTGCTGGACT
F275I	GCATTCGCTACCTCCATCGTCGTCTGCTGGACT
F275L	GCATTCGCTACCTCCCTGGTCGTCTGCTGGACT
F275K	GCATTCGCTACCTCCAAGGTCGTCTGCTGGACT
F275M	GCATTCGCTACCTCCATGGTCGTCTGCTGGACT
F275P	GCATTCGCTACCTCCCCCGTCGTCTGCTGGACT
F275S	GCATTCGCTACCTCCAGCGTCGTCTGCTGGACT
F275T	GCATTCGCTACCTCCACCGTCGTCTGCTGGACT
F275W	GCATTCGCTACCTCCTGGGTCGTCTGCTGGACT
F275Y	GCATTCGCTACCTCCTACGTCGTCTGCTGGACT
F275V	GCATTCGCTACCTCCGTGGTCGTCTGCTGGACT
V277A	GCTACCTCCTTTGTCGCCTGCTGGACTCCCTAC
V277R	GCTACCTCCTTTGTCAGATGCTGGACTCCCTAC

V277N	GCTACCTCCTTTGTCAACTGCTGGACTCCCTAC
V277D	GCTACCTCCTTTGTCGACTGCTGGACTCCCTAC
V277C	GCTACCTCCTTTGTCTGCTGCTGGACTCCCTAC
V277Q	GCTACCTCCTTTGTCCAGTGCTGGACTCCCTAC
V277E	GCTACCTCCTTTGTCGAGTGCTGGACTCCCTAC
V277G	GCTACCTCCTTTGTCGGCTGCTGGACTCCCTAC
V277H	GCTACCTCCTTTGTCCACTGCTGGACTCCCTAC
V277I	GCTACCTCCTTTGTCATCTGCTGGACTCCCTAC
V277L	GCTACCTCCTTTGTCCTGTGCTGGACTCCCTAC
V277K	GCTACCTCCTTTGTCAAGTGCTGGACTCCCTAC
V277M	GCTACCTCCTTTGTCATGTGCTGGACTCCCTAC
V277F	GCTACCTCCTTTGTCTTCTGCTGGACTCCCTAC
V277P	GCTACCTCCTTTGTCCCCTGCTGGACTCCCTAC
V277S	GCTACCTCCTTTGTCAGCTGCTGGACTCCCTAC
V277T	GCTACCTCCTTTGTCACCTGCTGGACTCCCTAC
V277W	GCTACCTCCTTTGTCTGGTGCTGGACTCCCTAC
V277Y	GCTACCTCCTTTGTCTACTGCTGGACTCCCTAC
C278A	ACCTCCTTTGTCGTCGCCTGGACTCCCTACTAT
C278R	ACCTCCTTTGTCGTCAGATGGACTCCCTACTAT
C278N	ACCTCCTTTGTCGTCAACTGGACTCCCTACTAT
C278D	ACCTCCTTTGTCGTCGACTGGACTCCCTACTAT
C278Q	ACCTCCTTTGTCGTCCAGTGGACTCCCTACTAT
C278E	ACCTCCTTTGTCGTCGAGTGGACTCCCTACTAT
C278G	ACCTCCTTTGTCGTCGGCTGGACTCCCTACTAT
C278H	ACCTCCTTTGTCGTCCACTGGACTCCCTACTAT
C278I	ACCTCCTTTGTCGTCATCTGGACTCCCTACTAT
C278L	ACCTCCTTTGTCGTCCTGTGGACTCCCTACTAT
C278K	ACCTCCTTTGTCGTCAAGTGGACTCCCTACTAT
C278M	ACCTCCTTTGTCGTCATGTGGACTCCCTACTAT
C278F	ACCTCCTTTGTCGTCTTCTGGACTCCCTACTAT
C278P	ACCTCCTTTGTCGTCCCCTGGACTCCCTACTAT
C278S	ACCTCCTTTGTCGTCAGCTGGACTCCCTACTAT
C278T	ACCTCCTTTGTCGTCACCTGGACTCCCTACTAT
C278W	ACCTCCTTTGTCGTCTGGTGGACTCCCTACTAT
C278Y	ACCTCCTTTGTCGTCTACTGGACTCCCTACTAT
C278V	ACCTCCTTTGTCGTCGTGTGGACTCCCTACTAT
L285A	ACTCCCTACTATGTCGCCGGCATTTGGTACTGG
L285R	ACTCCCTACTATGTCAGAGGCATTTGGTACTGG
L285N	ACTCCCTACTATGTCAACGGCATTTGGTACTGG
L285D	ACTCCCTACTATGTCGACGGCATTTGGTACTGG
L285C	ACTCCCTACTATGTCTGCGGCATTTGGTACTGG

L285Q	ACTCCCTACTATGTCCAGGGCATTTGGTACTGG
L285E	ACTCCCTACTATGTCGAGGGCATTTGGTACTGG
L285G	ACTCCCTACTATGTCGGCGGCATTTGGTACTGG
L285H	ACTCCCTACTATGTCCACGGCATTTGGTACTGG
L285I	ACTCCCTACTATGTCATCGGCATTTGGTACTGG
L285K	ACTCCCTACTATGTCAAGGGCATTTGGTACTGG
L285M	ACTCCCTACTATGTCATGGGCATTTGGTACTGG
L285F	ACTCCCTACTATGTCTTCGGCATTTGGTACTGG
L285P	ACTCCCTACTATGTCCCCGGCATTTGGTACTGG
L285S	ACTCCCTACTATGTCAGCGGCATTTGGTACTGG
L285T	ACTCCCTACTATGTCACCGGCATTTGGTACTGG
L285W	ACTCCCTACTATGTCTGGGGCATTTGGTACTGG
L285Y	ACTCCCTACTATGTCTACGGCATTTGGTACTGG
L285V	ACTCCCTACTATGTCGTGGGCATTTGGTACTGG
W288A	TATGTCCTAGGCATTGCCTACTGGTTTGATCCA
W288R	TATGTCCTAGGCATTAGATACTGGTTTGATCCA
W288N	TATGTCCTAGGCATTAACTACTGGTTTGATCCA
W288D	TATGTCCTAGGCATTGACTACTGGTTTGATCCA
W288C	TATGTCCTAGGCATTTGCTACTGGTTTGATCCA
W288Q	TATGTCCTAGGCATTCAGTACTGGTTTGATCCA
W288E	TATGTCCTAGGCATTGAGTACTGGTTTGATCCA
W288G	TATGTCCTAGGCATTGGCTACTGGTTTGATCCA
W288H	TATGTCCTAGGCATTCACTACTGGTTTGATCCA
W288I	TATGTCCTAGGCATTATCTACTGGTTTGATCCA
W288L	TATGTCCTAGGCATTCTGTACTGGTTTGATCCA
W288K	TATGTCCTAGGCATTAAGTACTGGTTTGATCCA
W288M	TATGTCCTAGGCATTATGTACTGGTTTGATCCA
W288F	TATGTCCTAGGCATTTTCTACTGGTTTGATCCA
W288P	TATGTCCTAGGCATTCCCTACTGGTTTGATCCA
W288S	TATGTCCTAGGCATTAGCTACTGGTTTGATCCA
W288T	TATGTCCTAGGCATTACCTACTGGTTTGATCCA
W288Y	TATGTCCTAGGCATTTACTACTGGTTTGATCCA
W288V	TATGTCCTAGGCATTGTGTACTGGTTTGATCCA
M295A	TGGTTTGATCCAGAAGCCTTGAACAGGGTGTCA
M295R	TGGTTTGATCCAGAAAGATTGAACAGGGTGTCA
M295N	TGGTTTGATCCAGAAAACTTGAACAGGGTGTCA
M295D	TGGTTTGATCCAGAAGACTTGAACAGGGTGTCA
M295C	TGGTTTGATCCAGAATGCTTGAACAGGGTGTCA
M295Q	TGGTTTGATCCAGAACAGTTGAACAGGGTGTCA
M295E	TGGTTTGATCCAGAAGAGTTGAACAGGGTGTCA
M295G	TGGTTTGATCCAGAAGGCTTGAACAGGGTGTCA

M295H	TGGTTTGATCCAGAACACTTGAACAGGGTGTCA
M295I	TGGTTTGATCCAGAAATCTTGAACAGGGTGTCA
M295L	TGGTTTGATCCAGAACTGTTGAACAGGGTGTCA
M295K	TGGTTTGATCCAGAAAAGTTGAACAGGGTGTCA
M295F	TGGTTTGATCCAGAATTCTTGAACAGGGTGTCA
M295P	TGGTTTGATCCAGAACCCTTGAACAGGGTGTCA
M295S	TGGTTTGATCCAGAAAGCTTGAACAGGGTGTCA
M295T	TGGTTTGATCCAGAAACCTTGAACAGGGTGTCA
M295W	TGGTTTGATCCAGAATGGTTGAACAGGGTGTCA
M295Y	TGGTTTGATCCAGAATACTTGAACAGGGTGTCA
M295V	TGGTTTGATCCAGAAGTGTTGAACAGGGTGTCA
V299A	GAAATGTTGAACAGGGCCTCAGAGCCAGTGAAT
V299R	GAAATGTTGAACAGGAGATCAGAGCCAGTGAAT
V299N	GAAATGTTGAACAGGAACTCAGAGCCAGTGAAT
V299D	GAAATGTTGAACAGGGACTCAGAGCCAGTGAAT
V299C	GAAATGTTGAACAGGTGCTCAGAGCCAGTGAAT
V299Q	GAAATGTTGAACAGGCAGTCAGAGCCAGTGAAT
V299E	GAAATGTTGAACAGGGAGTCAGAGCCAGTGAAT
V299G	GAAATGTTGAACAGGGGCTCAGAGCCAGTGAAT
V299H	GAAATGTTGAACAGGCACTCAGAGCCAGTGAAT
V299I	GAAATGTTGAACAGGATCTCAGAGCCAGTGAAT
V299L	GAAATGTTGAACAGGCTGTCAGAGCCAGTGAAT
V299K	GAAATGTTGAACAGGAAGTCAGAGCCAGTGAAT
V299M	GAAATGTTGAACAGGATGTCAGAGCCAGTGAAT
V299F	GAAATGTTGAACAGGTTCTCAGAGCCAGTGAAT
V299P	GAAATGTTGAACAGGCCCTCAGAGCCAGTGAAT
V299S	GAAATGTTGAACAGGAGCTCAGAGCCAGTGAAT
V299T	GAAATGTTGAACAGGACCTCAGAGCCAGTGAAT
V299W	GAAATGTTGAACAGGTGGTCAGAGCCAGTGAAT
V299Y	GAAATGTTGAACAGGTACTCAGAGCCAGTGAAT
V303A	AGGGTGTCAGAGCCAGCCAATCACTTTTTCTTT
V303R	AGGGTGTCAGAGCCAAGAAATCACTTTTTCTTT
V303N	AGGGTGTCAGAGCCAAACAATCACTTTTTCTTT
V303D	AGGGTGTCAGAGCCAGACAATCACTTTTTCTTT
V303C	AGGGTGTCAGAGCCATGCAATCACTTTTTCTTT
V303Q	AGGGTGTCAGAGCCACAGAATCACTTTTTCTTT
V303E	AGGGTGTCAGAGCCAGAGAATCACTTTTTCTTT
V303G	AGGGTGTCAGAGCCAGGCAATCACTTTTTCTTT
V303H	AGGGTGTCAGAGCCACACAATCACTTTTTCTTT
V303I	AGGGTGTCAGAGCCAATCAATCACTTTTTCTTT
V303L	AGGGTGTCAGAGCCACTGAATCACTTTTTCTTT

V303K	AGGGTGTCAGAGCCAAAGAATCACTTTTTCTTT
V303M	AGGGTGTCAGAGCCAATGAATCACTTTTTCTTT
V303F	AGGGTGTCAGAGCCATTCAATCACTTTTTCTTT
V303P	AGGGTGTCAGAGCCACCCAATCACTTTTTCTTT
V303S	AGGGTGTCAGAGCCAAGCAATCACTTTTTCTTT
V303T	AGGGTGTCAGAGCCAACCAATCACTTTTTCTTT
V303W	AGGGTGTCAGAGCCATGGAATCACTTTTTCTTT
V303Y	AGGGTGTCAGAGCCATACAATCACTTTTTCTTT
F310A	CACTTTTTCTTTCTCGCCGCTTTCCTAAACCCG
F310R	CACTTTTTCTTTCTCAGAGCTTTCCTAAACCCG
F310N	CACTTTTTCTTTCTCAACGCTTTCCTAAACCCG
F310D	CACTTTTTCTTTCTCGACGCTTTCCTAAACCCG
F310C	CACTTTTTCTTTCTCTGCGCTTTCCTAAACCCG
F310Q	CACTTTTTCTTTCTCCAGGCTTTCCTAAACCCG
F310E	CACTTTTTCTTTCTCGAGGCTTTCCTAAACCCG
F310G	CACTTTTTCTTTCTCGGCGCTTTCCTAAACCCG
F310H	CACTTTTTCTTTCTCCACGCTTTCCTAAACCCG
F310I	CACTTTTTCTTTCTCATCGCTTTCCTAAACCCG
F310L	CACTTTTTCTTTCTCCTGGCTTTCCTAAACCCG
F310K	CACTTTTTCTTTCTCAAGGCTTTCCTAAACCCG
F310M	CACTTTTTCTTTCTCATGGCTTTCCTAAACCCG
F310P	CACTTTTTCTTTCTCCCCGCTTTCCTAAACCCG
F310S	CACTTTTTCTTTCTCAGCGCTTTCCTAAACCCG
F310T	CACTTTTTCTTTCTCACCGCTTTCCTAAACCCG
F310W	CACTTTTTCTTTCTCTGGGCTTTCCTAAACCCG
F310Y	CACTTTTTCTTTCTCTACGCTTTCCTAAACCCG
F310V	CACTTTTTCTTTCTCGTGGCTTTCCTAAACCCG
A311R	TTTTTCTTTCTCTTTAGATTCCTAAACCCGTGC
A311N	TTTTTCTTTCTCTTTAACTTCCTAAACCCGTGC
A311D	TTTTTCTTTCTCTTTGACTTCCTAAACCCGTGC
A311C	TTTTTCTTTCTCTTTTGCTTCCTAAACCCGTGC
A311Q	TTTTTCTTTCTCTTTCAGTTCCTAAACCCGTGC
A311E	TTTTTCTTTCTCTTTGAGTTCCTAAACCCGTGC
A311G	TTTTTCTTTCTCTTTGGCTTCCTAAACCCGTGC
A311H	TTTTTCTTTCTCTTTCACTTCCTAAACCCGTGC
A311I	TTTTTCTTTCTCTTTATCTTCCTAAACCCGTGC
A311L	TTTTTCTTTCTCTTTCTGTTCCTAAACCCGTGC
A311K	TTTTTCTTTCTCTTTAAGTTCCTAAACCCGTGC
A311M	TTTTTCTTTCTCTTTATGTTCCTAAACCCGTGC
A311F	TTTTTCTTTCTCTTTTTCTTCCTAAACCCGTGC
A311P	TTTTTCTTTCTCTTTCCCTTCCTAAACCCGTGC

A311S	TTTTTCTTTCTCTTTAGCTTCCTAAACCCGTGC
A311T	TTTTTCTTTCTCTTTACCTTCCTAAACCCGTGC
A311W	TTTTTCTTTCTCTTTTGGTTCCTAAACCCGTGC
A311Y	TTTTTCTTTCTCTTTTACTTCCTAAACCCGTGC
A311V	TTTTTCTTTCTCTTTGTGTTCCTAAACCCGTGC
F317A	TTCCTAAACCCGTGCGCCGACCCACTCATATAT
F317R	TTCCTAAACCCGTGCAGAGACCCACTCATATAT
F317N	TTCCTAAACCCGTGCAACGACCCACTCATATAT
F317D	TTCCTAAACCCGTGCGACGACCCACTCATATAT
F317C	TTCCTAAACCCGTGCTGCGACCCACTCATATAT
F317Q	TTCCTAAACCCGTGCCAGGACCCACTCATATAT
F317E	TTCCTAAACCCGTGCGAGGACCCACTCATATAT
F317G	TTCCTAAACCCGTGCGGCGACCCACTCATATAT
F317H	TTCCTAAACCCGTGCCACGACCCACTCATATAT
F317I	TTCCTAAACCCGTGCATCGACCCACTCATATAT
F317L	TTCCTAAACCCGTGCCTGGACCCACTCATATAT
F317K	TTCCTAAACCCGTGCAAGGACCCACTCATATAT
F317M	TTCCTAAACCCGTGCATGGACCCACTCATATAT
F317P	TTCCTAAACCCGTGCCCCGACCCACTCATATAT
F317S	TTCCTAAACCCGTGCAGCGACCCACTCATATAT
F317T	TTCCTAAACCCGTGCACCGACCCACTCATATAT
F317W	TTCCTAAACCCGTGCTGGGACCCACTCATATAT
F317Y	TTCCTAAACCCGTGCTACGACCCACTCATATAT
F317V	TTCCTAAACCCGTGCGTGGACCCACTCATATAT
D318A	CTAAACCCGTGCTTCGCCCCACTCATATATGGG
D318R	CTAAACCCGTGCTTCAGACCACTCATATATGGG
D318N	CTAAACCCGTGCTTCAACCCACTCATATATGGG
D318C	CTAAACCCGTGCTTCTGCCCACTCATATATGGG
D318Q	CTAAACCCGTGCTTCCAGCCACTCATATATGGG
D318E	CTAAACCCGTGCTTCGAGCCACTCATATATGGG
D318G	CTAAACCCGTGCTTCGGCCCACTCATATATGGG
D318H	CTAAACCCGTGCTTCCACCCACTCATATATGGG
D318I	CTAAACCCGTGCTTCATCCCACTCATATATGGG
D318L	CTAAACCCGTGCTTCCTGCCACTCATATATGGG
D318K	CTAAACCCGTGCTTCAAGCCACTCATATATGGG
D318M	CTAAACCCGTGCTTCATGCCACTCATATATGGG
D318F	CTAAACCCGTGCTTCTTCCCACTCATATATGGG
D318P	CTAAACCCGTGCTTCCCCCCACTCATATATGGG
D318S	CTAAACCCGTGCTTCAGCCCACTCATATATGGG
D318T	CTAAACCCGTGCTTCACCCCACTCATATATGGG
D318W	CTAAACCCGTGCTTCTGGCCACTCATATATGGG

D318Y	CTAAACCCGTGCTTCTACCCACTCATATATGGG
D318V	CTAAACCCGTGCTTCGTGCCACTCATATATGGG
L320A	CCGTGCTTCGACCCAGCCATATATGGGTATTTC
L320R	CCGTGCTTCGACCCAAGAATATATGGGTATTTC
L320N	CCGTGCTTCGACCCAAACATATATGGGTATTTC
L320D	CCGTGCTTCGACCCAGACATATATGGGTATTTC
L320C	CCGTGCTTCGACCCATGCATATATGGGTATTTC
L320Q	CCGTGCTTCGACCCACAGATATATGGGTATTTC
L320E	CCGTGCTTCGACCCAGAGATATATGGGTATTTC
L320G	CCGTGCTTCGACCCAGGCATATATGGGTATTTC
L320H	CCGTGCTTCGACCCACACATATATGGGTATTTC
L320I	CCGTGCTTCGACCCAATCATATATGGGTATTTC
L320K	CCGTGCTTCGACCCAAAGATATATGGGTATTTC
L320M	CCGTGCTTCGACCCAATGATATATGGGTATTTC
L320F	CCGTGCTTCGACCCATTCATATATGGGTATTTC
L320P	CCGTGCTTCGACCCACCCATATATGGGTATTTC
L320S	CCGTGCTTCGACCCAAGCATATATGGGTATTTC
L320T	CCGTGCTTCGACCCAACCATATATGGGTATTTC
L320W	CCGTGCTTCGACCCATGGATATATGGGTATTTC
L320Y	CCGTGCTTCGACCCATACATATATGGGTATTTC
L320V	CCGTGCTTCGACCCAGTGATATATGGGTATTTC
Y322A	TTCGACCCACTCATAGCCGGGTATTTCTCTTTG
Y322R	TTCGACCCACTCATAAGAGGGTATTTCTCTTTG
Y322N	TTCGACCCACTCATAAACGGGTATTTCTCTTTG
Y322D	TTCGACCCACTCATAGACGGGTATTTCTCTTTG
Y322C	TTCGACCCACTCATATGCGGGTATTTCTCTTTG
Y322Q	TTCGACCCACTCATACAGGGGTATTTCTCTTTG
Y322E	TTCGACCCACTCATAGAGGGGTATTTCTCTTTG
Y322G	TTCGACCCACTCATAGGCGGGTATTTCTCTTTG
Y322H	TTCGACCCACTCATACACGGGTATTTCTCTTTG
Y322I	TTCGACCCACTCATAATCGGGTATTTCTCTTTG
Y322L	TTCGACCCACTCATACTGGGGTATTTCTCTTTG
Y322K	TTCGACCCACTCATAAAGGGGTATTTCTCTTTG
Y322M	TTCGACCCACTCATAATGGGGTATTTCTCTTTG
Y322F	TTCGACCCACTCATATTCGGGTATTTCTCTTTG
Y322P	TTCGACCCACTCATACCCGGGTATTTCTCTTTG
Y322S	TTCGACCCACTCATAAGCGGGTATTTCTCTTTG
Y322T	TTCGACCCACTCATAACCGGGTATTTCTCTTTG
Y322W	TTCGACCCACTCATATGGGGGTATTTCTCTTTG
Y322V	TTCGACCCACTCATAGTGGGGTATTTCTCTTTG
L327A	TATGGGTATTTCTCTGCCTAGGGCGGCCGCTCG

L327R	TATGGGTATTTCTCTAGATAGGGCGGCCGCTCG
L327N	TATGGGTATTTCTCTAACTAGGGCGGCCGCTCG
L327D	TATGGGTATTTCTCTGACTAGGGCGGCCGCTCG
L327C	TATGGGTATTTCTCTTGCTAGGGCGGCCGCTCG
L327Q	TATGGGTATTTCTCTCAGTAGGGCGGCCGCTCG
L327E	TATGGGTATTTCTCTGAGTAGGGCGGCCGCTCG
L327G	TATGGGTATTTCTCTGGCTAGGGCGGCCGCTCG
L327H	TATGGGTATTTCTCTCACTAGGGCGGCCGCTCG
L327I	TATGGGTATTTCTCTATCTAGGGCGGCCGCTCG
L327K	TATGGGTATTTCTCTAAGTAGGGCGGCCGCTCG
L327M	TATGGGTATTTCTCTATGTAGGGCGGCCGCTCG
L327F	TATGGGTATTTCTCTTTCTAGGGCGGCCGCTCG
L327P	TATGGGTATTTCTCTCCCTAGGGCGGCCGCTCG
L327S	TATGGGTATTTCTCTAGCTAGGGCGGCCGCTCG
L327T	TATGGGTATTTCTCTACCTAGGGCGGCCGCTCG
L327W	TATGGGTATTTCTCTTGGTAGGGCGGCCGCTCG
L327Y	TATGGGTATTTCTCTTACTAGGGCGGCCGCTCG
L327V	TATGGGTATTTCTCTGTGTAGGGCGGCCGCTCG
